# The Bark Beetle *Dendroctonus rhizophagus* (Curculionidae: Scolytinae) Has Digestive Capacity to Degrade Complex Substrates: Functional Characterization and Heterologous Expression of an α-Amylase

**DOI:** 10.3390/ijms22010036

**Published:** 2020-12-22

**Authors:** L. Viridiana Soto-Robles, María Fernanda López, Verónica Torres-Banda, Claudia Cano-Ramírez, Gabriel Obregón-Molina, Gerardo Zúñiga

**Affiliations:** Laboratorio de Variación Biológica y Evolución, Departamento de Zoología, Escuela Nacional de Ciencias Biológicas, Instituto Politécnico Nacional, Prolongación de Carpio y Plan de Ayala s/n, Santo Tomás, Miguel Hidalgo, Mexico City 11340, Mexico; lviridianasr@gmail.com (L.V.S.-R.); mariferlopez@hotmail.com (M.F.L.); vtbanda@yahoo.com.mx (V.T.-B.); clacanram@yahoo.com.mx (C.C.-R.); gobregonm@live.com (G.O.-M.)

**Keywords:** α-amylase, *Dendroctonus*, bark beetle, heterologous expression, RT-qPCR, modelling

## Abstract

*Dendroctonus*-bark beetles are natural agents contributing to vital processes in coniferous forests, such as regeneration, succession, and material recycling, as they colonize and kill damaged, stressed, or old pine trees. These beetles spend most of their life cycle under stem and roots bark where they breed, develop, and feed on phloem. This tissue is rich in essential nutrients and complex molecules such as starch, cellulose, hemicellulose, and lignin, which apparently are not available for these beetles. We evaluated the digestive capacity of *Dendroctonus rhizophagus* to hydrolyze starch. Our aim was to identify α-amylases and characterize them both molecularly and biochemically. The findings showed that *D. rhizophagus* has an α-amylase gene (*AmyDr*) with a single isoform, and ORF of 1452 bp encoding a 483-amino acid protein (53.15 kDa) with a predicted signal peptide of 16 amino acids. AmyDr has a mutation in the chlorine-binding site, present in other phytophagous insects and in a marine bacterium. Docking analysis showed that AmyDr presents a higher binding affinity to amylopectin compared to amylose, and an affinity binding equally stable to calcium, chlorine, and nitrate ions. AmyDr native protein showed amylolytic activity in the head-pronotum and gut, and its recombinant protein, a polypeptide of ~53 kDa, showed conformational stability, and its activity is maintained both in the presence and absence of chlorine and nitrate ions. The *AmyDr* gene showed a differential expression significantly higher in the gut than the head-pronotum, indicating that starch hydrolysis occurs mainly in the midgut. An overview of the *AmyDr* gene expression suggests that the amylolytic activity is regulated through the developmental stages of this bark beetle and associated with starch availability in the host tree.

## 1. Introduction

*Dendroctonus*-bark beetles (Curculionidae: Scolytinae) play a fundamental role in the structure of coniferous forests, because they colonize and kill sick, damaged, and weakened trees, contributing to community succession, nutrient cycling, and canopy thinning [1]. However, some of their species are also disturbance agents because, under certain forest stress conditions, outbreaks occur that affect hundreds of thousands of conifer trees of the genera *Larix*, *Picea*, *Pinus*, and *Pseudotsuga* from North and Central America [2,3,4].

*Dendroctonus*-bark beetles are phytophagous insects that spend most of their life cycle under the bark of host plants, where they reproduce, feed on phloem, and interact with microbial symbionts and invertebrates. These symbionts have been isolated from the exoskeleton, galleries, and gut of these bark beetles [5,6,7,8,9,10]. A core gut bacteriome of *Dendroctonus* species is known [10], as well as the yeasts, mites, and nematodes diversity associated to them [11]. Some of microbial symbionts are capable of degrading different substrates such as starch, esters, and lipids [12], cellulose [13], recycling uric acid [7], and degrading or transforming different monoterpenes from the host trees of these insects [14,15,16].

The phloem is a conduction system that transports different molecules and ions such as mRNAs, hormones, sugars, and nutrients such as trace elements and amino acids towards different plant body parts. As constitutive tissue is formed by different cell types (e.g., sieve cells and sieve tube elements integrating the conduction system itself, the companion and parenchyma cells), which represent a substrate rich in organic acids and non-structural carbohydrates (e.g., starch, sucrose, raffinose, stachyose, verbascose), as well as in structural carbohydrates (e.g., cellulose, hemicellulose). In particular, starch is the main carbohydrate reserve of the plants, synthesized from sugars produced during photosynthesis both in autotrophic and heterotrophic tissues (e.g., roots, woody tissues, fruits, seeds, tubers, and pollen grains) [17].

Starch is stored in plants as insoluble particles or granules and is composed of amylose and amylopectin [18]. Amylose constitutes about ~25% of starch and is essentially linear with α-D (1→4) linked glucose units and a few branched points per molecule. Amylopectin is highly branched and constitutes ~75% of starch, it is a polymer of α-D (1→4) linked glucose units and joined by α-D (1→6) linkages after every 24–30 glucose units [19]. The structural complexity of starch requires different mechanisms for its hydrolysis, where enzymes such as α-amylases (α-1,4-glucan-4-glucanohydrolases; EC 3.2.1.1) are fundamental to catalyze this polysaccharide into low-molecular-weight molecules and other carbohydrates as well as α-dextrins, maltotriose, and maltose [20,21,22]. Given that starch is the most abundant polysaccharide reserve in different plant tissues, it has been hypothesized that products derived from its hydrolysis might be utilized as essential food sources by insects for their development and survival. Several studies have reported that the number of α-amylase gene copies (from 1 to 13) is variable in insects. Some of them have been biochemically characterized, sequenced, and their phylogenetic relationship inferred, as well as their location, enzyme excretion sites, and regulatory mechanisms [23]. Unfortunately, in bark beetles, α-amylases have received very little attention. Studies in population genetics have demonstrated the presence of allelic variants of these enzymes [24]. However, Viktorinova et al. [25] demonstrated in *I. typographus* the presence of two α-amylase genes one of them producing two isoforms as a result of alternative splicing.

In this study, we analyzed the α-amylases of *Dendroctonus rhizophagus* Thomas & Bright, an endemic species to the Sierra Madre Occidental in Mexico, which colonizes and kills seedlings and saplings (<3 m height and ~10 cm diameter) of several pine species [26,27]. The life cycle of *D. rhizophagus* is annual, univoltine, and atypical among *Dendroctonus* species. This species does not perform massive attacks on trees. Just one pair of insects colonizes and kills individual trees. In particular, we molecularly characterized the enzyme AmyDr, determined its number of isoforms, analyzed the relative expression of α-amylase *AmyDr* gene through different developmental stages, provided functional evidence that both native and recombinant α-amylase AmyDr of this bark beetle are capable of hydrolyzing starch, and determined the effect of metal and non-metal ions on recombinant α-amylase activity.

## 2. Results

### 2.1. In Silico Molecular Characterization

A single α-amylase gene was identified, cloned, and sequenced. All the sequenced clones showed a pairwise nucleotide identity ~98%, nucleotide substitutions were mainly synonymous and a few no synonymous. The mapping of the cDNA sequences of this gene against the transcriptome of *D. rhizophagus* identified a single transcript that was annotated as an α-amylase. Therefore, isoforms of this protein were not present in *D. rhizophagus*. The α-amylase gene (the protein hereafter named AmyDr) had an open reading frame of 1452 bp that encodes 483 amino acid residues with a predicted N-terminal signal peptide of 16 amino acid residues. AmyDr has seven potential O-glycosylation sites and one single potential N-glycosylation site, a predicted molecular mass of around 53.15 kDa, theoretical pI of 4.54, and extracellular localization.

**Figure 1:**
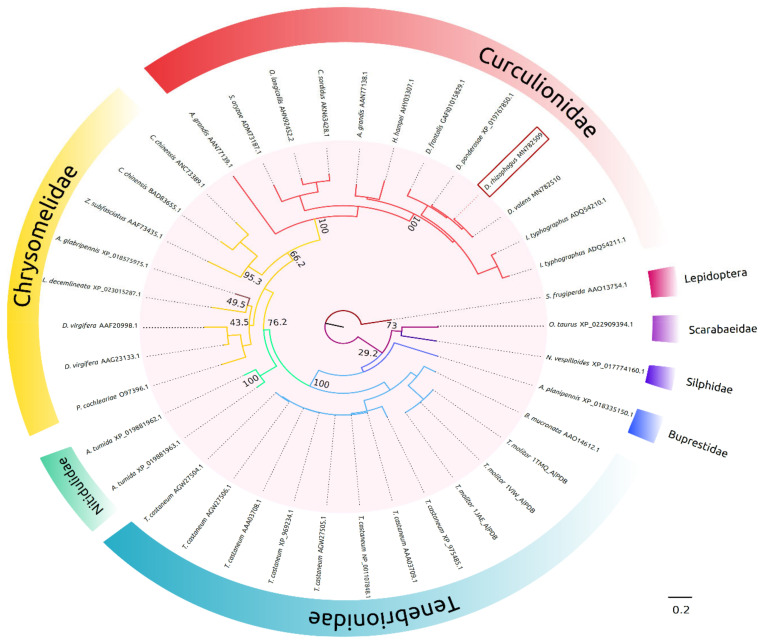
Maximum-likelihood tree of α-amylases from coleopteran amino acid sequences of *D. rhizophagus* and GenBank and PDB sequences. The analysis was performed using the amino acid substitution model LG + G + I + F; G = 1.355, I = 0.141. The accession numbers of GenBank or PDB sequences are shown at the end of each branch, bootstrap values after 1000 pseudoreplicates are shown at nodes. AmyDr is boxed in red. The α-amylase sequence of *Spodoptera frugiperda* (AAO13754.1) was used as outgroup.

Phylogenetic inference analysis by maximum likelihood (Figure 1) showed the formation of different α-amylase groups, all of which were well supported (bootstrap value > 60%) and according to the insect families. In the Curculionidae family, the α-amylases of *Dendroctonus* species formed a consistent (bootstrap value = 100%) and monophyletic group, different from other scolytines, as is the case of *Ips* and *Hypothenemus* genera. AmyDr had a mean amino acid identity >90% with α-amylases of other *Dendroctonus* species and an identity of around 80% with α-amylases of scolytines. Three chrysomelid sequences of species *Callosobruchus chinensis* and *Zabrotes subfasciatus* were clustered into the α-amylases group from Curculionidae, which share in the Cl-binding site the substitution of lysine by arginine with scolytines.

**Figure 2:**
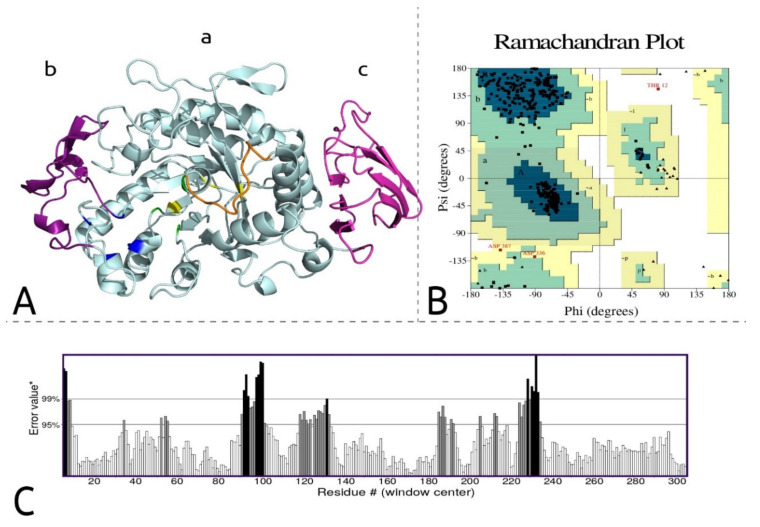
Structural model of the α-amylase of *D. rhizophagus*: AmyDr. (**A**) The cartoon structure: Letters a, b, and c indicate α-amylase domains, the arrows indicate β-sheet structures, helical structures indicate α-helices, the flexible loop is coloured in orange, the catalytic site is indicated in green, the calcium binding site in blue, and chlorine binding site in yellow. (**B**) Ramachandran plot: residues in the most favoured regions (A, B, L), residues in additional allowed regions (a, b, l, p), residues in generously allowed regions (~a, ~b, ~l, ~p), residues in disallowed regions (white region). (**C**) Error-values* plotted by Errat: the error function is based on the statistics of non-bonded atom-atom interactions in the structure.

AmyDr shares an amino acid identity of 55% with the crystallized α-amylase of *Tenebrio molitor* (TMA). The structural model of AmyDr presented 97.64% of the amino acids with a score ≥0.2 in the 3D/1D profile and an average overall quality factor of 81.9172. The analysis of torsional angles Phi and Psi showed 91.9% (372) of residues in the most favoured regions, 7.4% (30) residues in additional allowed regions, 0.5% (two) residues in generously allowed regions, and 0.2% (one) residues in the disallowed regions (Figure 2). Moreover, the alignment of the AmyDr model against the TMA model showed a value of RMSD = 0.30, suggesting that the structure of this α-amylase is highly conserved.

The structural model of AmyDr (Figure 2) indicated that this protein presents three domains (A, B, and C) commonly recognized in animal α-amylases. Domain A is a TIM barrel (α/β)_8_, Domain B is represented by two short β-sheets, and Domain C is known as the Greek key domain formed from an antiparallel β-sheet set. In particular, domain A or the catalytic domain has highly conserved residues that are involved in enzyme catalysis, these residues are part of the active site and form a catalytic triad (AmyDr: ASP184, GLU220, ASP285). Also, this domain has binding sites to calcium ion (AmyDr: ASN98, ARG145, ASP154, HIS188) and binding sites to chlorine ion (AmyDr: ARG182, ASN283, LYS319). The overall alignment of AmyDr and α-amylases of other coleopterans showed that the residues involved in the catalytic process and binding site to calcium are highly conserved. However, in the binding site to chlorine ion, AmyDr has one residue mutated (ARG to LYS) in position 319 (= position 321 in Supplementary Figure S1) similar to α-amylases from other curculionids; chrysomelids, and hymenopterans.

**Figure 3:**
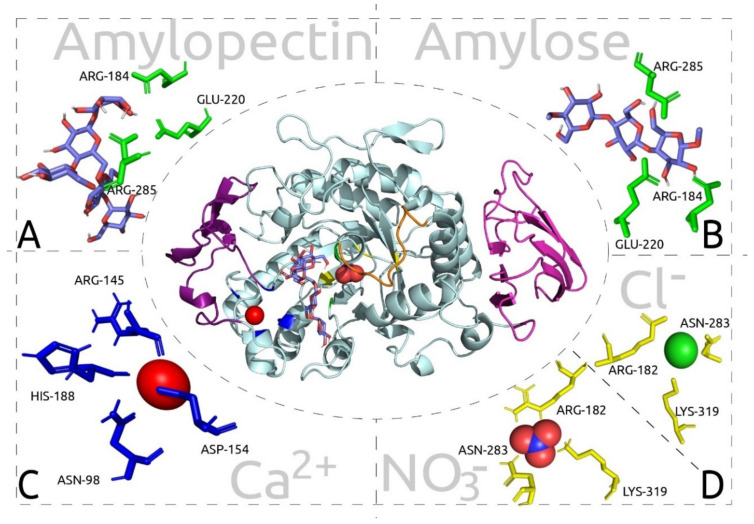
Predicted docked interactions of AmyDr protein from *D. rhizophagus.* Cartoon diagram highlighting the interaction with ligands is shown to the centre. (**A**) Ligand: amylopectin. (**B**) Ligand: amylose. (**C**) Ligand: calcium ion. (**D**) Ligand: nitrate and chlorine ions.

Docking analyses showed that residues ASP184, GLU220, and ASP285 present an association or binding affinity with amylopectin with a binding energy of −33.72 kJ mol^−1^ (Figure 3A), but less to amylose, with a binding energy of −23.01 kJ mol^−1^ (Figure 3B). A stable binding affinity of the calcium ion was also observed between residues ASN98, ARG145, ASP154, and HIS 188 of AmyDr, with a binding energy of −34.27 kJ mol^−1^ (Figure 3C). Given the presence of LYS319 instead of ARG the docking analyses with Cl^−^ and NO_3_^−^ showed a strong binding affinity between these ligands and ARG182, ASN283, LYS319 residues (Figure 3D), with a binding energy of −16.75 and −17.2 kJ mol^−1^ and hydrogen bonds with distances ≤2.1 Å and 2.2 Å, respectively.

### 2.2. Assays of Functional Activity

**Figure 4:**
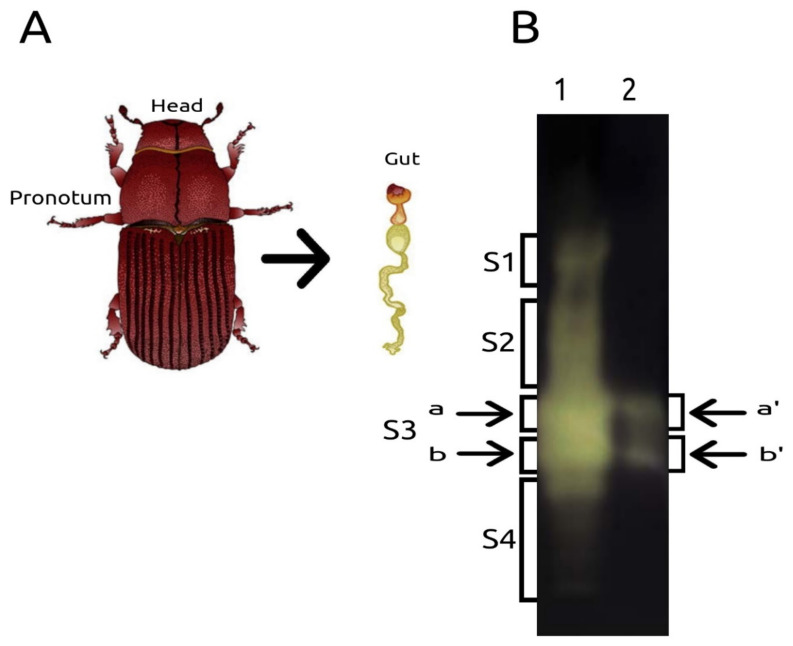
Zymogram of amylolytic activity. (**A**) scheme of *D. rhizophagus* indicating head-pronotum and gut samples. (**B**) amylolytic activity visualized in the native-PAGE. The gut sample was loaded in lane 1, while the head-pronotum sample was loaded in lane 2. All sections (S1–S4) were analyzed in the MALDI-TOF/TOF. The arrows indicate the subsections within S3 from gut (**a**,**b**) and head-pronotum (**a’**,**b’**) lanes analyzed.

**Figure 5:**
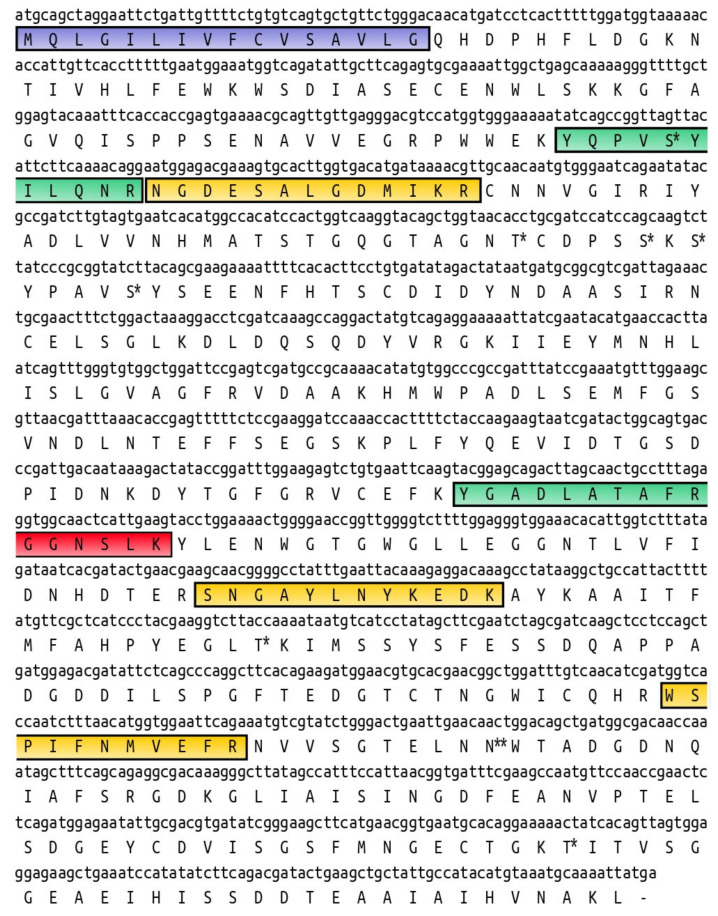
AmyDr cDNA and translation sequence. Identification of peptides. Peptides identified by MALDI-TOF/TOF in S2 and subsections a, b from the gut and a’, b’ from the head-pronotum are boxed in green. Peptides identified in S2 and only in a subsection a from S3 are boxed in yellow, peptides identified only in the section b from S3 are boxed in red. The predicted signal P is boxed in blue. Potential O-glycosylation sites are indicated with the symbol *. A potential N-glycosylation site is indicated with the symbol **.

Different patterns of amylolytic activity in native-PAGE were observed in the head-pronotum and gut samples of *D. rhizophagus* adults (Figure 4A–B). The amylolytic activity was evident in the four gut Sections (S1–S4), but it was major in S3 both gut and head-pronotum samples (Figure 4B). The MALDI-TOF/TOF allowed the identification and confirmation of AmyDr peptides only in S2 from the gut and in both subsections (a,b; a’b’) from S3 from head-pronotum and gut samples (Figure 5). AmyDr peptides were not recovered in S1 and S4 from the gut.

### 2.3. RT-qPCR Expression of AmyDr

**Figure 6:**
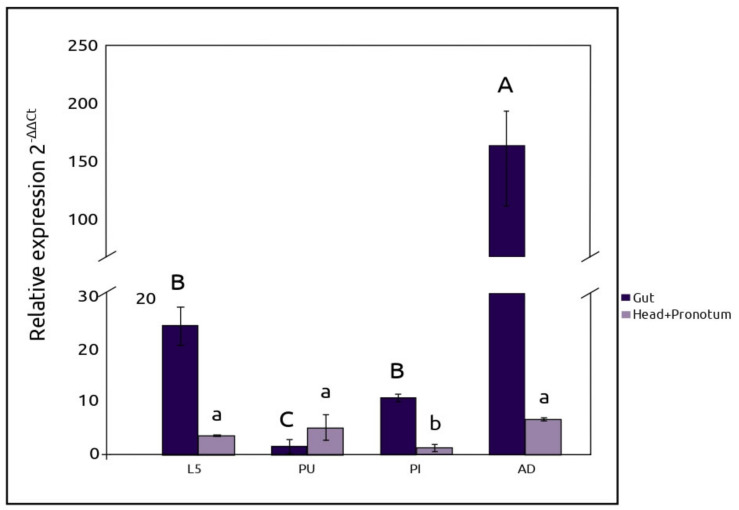
Relative expression of the *AmyDr* gene. The expression pattern of the *AmyDr* gene was determined by RT-qPCR using cDNA from the fifth-instar larvae (L5), pupa (PU), preimage (PI), and pre-emergent adult (AD) of *D. rhizophagus*. *GAPDH* gene was used as a reference gene for RT-qPCR normalization and the expression profile was relative to the egg stage. The means of 2^−ΔΔCt^ were graphed, three technical replicates were performed for each biological replicate for each developmental stage. Biological replicates were integrated by a pool of ten guts or ten head-pronotum. Significant differences between states are indicated with letters and the standard error with bars.

The *AmyDr* gene showed an increase in its expression in tissues and developmental stages (Figure 6). Significant differences in gut expression levels were found with Welch’s ANOVA (*F* = 103.8, *df* = 15.74, *p* < 0.001), and the Tukey test indicated that differences were found among adult gut, pre-imago-larvae gut, and pupae gut (*p* < 0.05) (Figure 6). On the other hand, Welch’s ANOVA also showed significant differences in *AmyDr* gene expression of the head-pronotum (*F* = 124.4, *df* = 15.22, *p* < 0.001). These differences were focused only between the adult, larvae, and pupae vs. pre-imago (*p* < 0.05).

### 2.4. Expression of AmyDr Recombinant Enzyme

**Figure 7:**
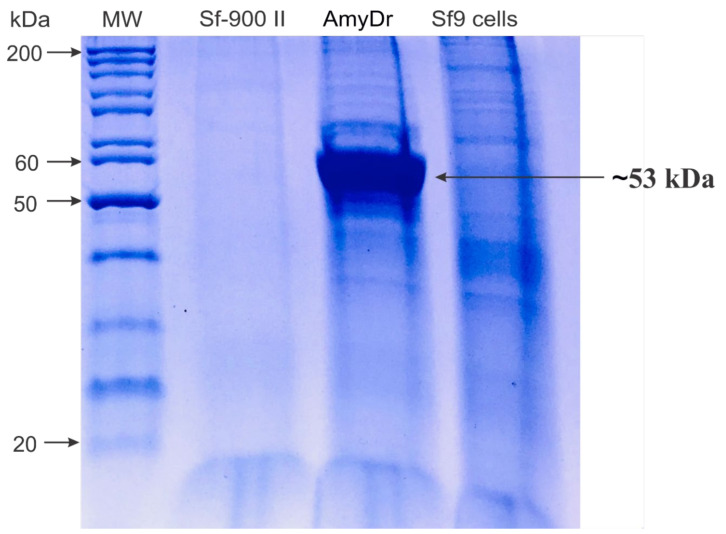
Analysis of total protein extracts from *Sf9*/Bac_*AmyDr* in 10% SDS-PAGE. Line 1, MW protein standards; line 2, Sf900-II culture medium; line 3, AmyDr, and line 4, (NH_4_)_2_SO_4_ precipitate from *Sf9* cells extract after dialysis. Lines 2 and 4, negative controls.

**Figure 8:**
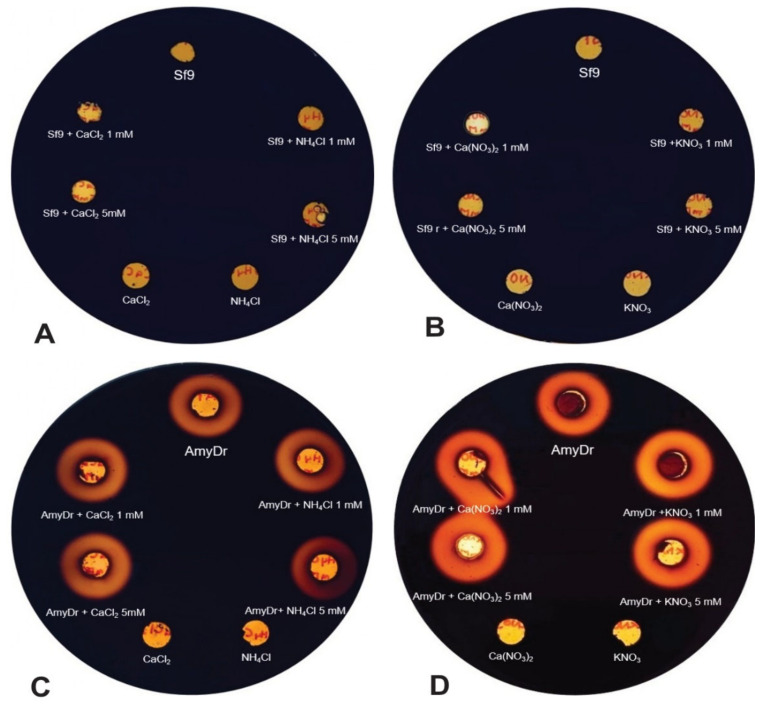
Effect of metal (Ca^2+^ and Cl^−1^) and non-metal ions (NO_3_^−1^) on AmyDr activity at two different concentrations (1 and 5 mM). (**A**,**B**) cells extract and (**C**,**D**) *Sf9* AmyDr, both incubated with CaCl_2_, NH_4_Cl, Ca(NO_3_)_2,_ and KNO_3_ (1 and 5 mM each) on 1% (*w*/*v*) starch agar plates during 12 h at 37 °C.

The profile of total protein in the supernatant of infected *Sf9* cells culture, compared to non-infected cells, showed the overexpression of a polypeptide with a molecular weight of ~53 kDa (Figure 7), which is close to the estimated value for amylase AmyDr. No amylolytic activity was observed in Sf9 cells (Figure 8A,B). The enzymatic activity of AmyDr was enhanced in the presence of Ca(NO_3_)_2_ and KNO_3_ at 1 and 5 mM, maintained also with CaCl_2_ and NH_4_Cl at both concentrations, and slightly with NH_4_Cl at 5 mM (Figure 8C,D).

## 3. Discussion

Our findings showed that AmyDr is a functional enzyme encoded by an ORF of 1452 nucleotides and is present both in the gut and head-pronotum of *Dendroctonus rhizophagus*. *In silico* analysis showed that this protein has a 51.51 kDa molecular mass (without signal peptide), similar to the α-amylase from other scolytines, such as *D. ponderosae* (51.37 kDa, XP_019767850.1), *Ips typographus* (51.84 kDa, ADQ54210.1; 52.19 kDa, ADQ54211.1), and *Hypothenemus hampei* (51.24 kDa, AHY03307), curculionids, such as *Antohonomus grandis* (50.87 kDa, AAN77138.1; 52.28 kDa, AAN77139.1) and *Sitophilus oryzae* (51.32 kDa, ADM73187.1), other coleopterans, such as *Tenebrio molitor* (51.24 kDa, P56634) and *Tribolium castaneum* (51.60 kDa, AAA03708), and other insects, such as *Spodoptera frugiperda* (54.78 kDa, AAO13754), *Drosophila melanogaster* (51.93 kDa, AAA92232), and *Apis mellifera* (54.25kDa, NP_001011598). The theoretical pI of AmyDr (4.54) is similar to the pI of coleopteran α-amylases (pI < 7) [23].

Only one AmyDr isoform is present in the gut and head-pronotum of *D. rhizophagus*, which agrees with the one found in other *Dendroctonus* species such as *D. valens* (MN782510), *D. frontalis* (GAFI01015829.1), and *D. ponderosae* (BT126692), and that reported in *H. hampei* and other curculionids such as *Odoiporus longicollis* (AHN92452.2) and *Cosmopolites sordidus* (AKN63428.1). However, this result is different from those found in *A. grandis*, *I. typographus*, and *Rhynchophorus ferrugineus* in which two *Amy* genes have been documented [25,28,29]. Based on the number of α-amylases genes (from 1 to 9) in Coleoptera [23], as well as on the bioinformatics analyses of transcriptomes and genomes from *Dendroctonus* species studied so far, the genus *Dendroctonus* presented the lowest number of α-amylases genes.

The sequence analysis of AmyDr clones showed mainly synonymous substitutions along the sequences, suggesting the presence of allelic variants of the protein. In addition, transcriptome analyses of *D. rhizophagus*, as well as from other *Dendroctonus* species and scolytines such as *D. ponderosae* (GAFW00000000), *D. frontalis* (GAFI00000000), *D. valens* [30], and *Tomicus yunnanensis* (GFJU00000000), referenced to *D. ponderosae* genome, did not show evidence of isoforms. The absence of isoforms in *Dendroctonus*-bark beetles suggests that the selection has favoured the occurrence of amylases with major functional plasticity, stability, and efficiency, but not the occurrence gene duplications. In contrast, in the bark beetle *I. typographus* two functional α-amylases have been reported, one of them includes two isoforms [25], an event rarely reported in animals because the common is the presence of multiple copies [23] by gene duplications [31] or horizontal transfer [32]. In fact, a gene duplication event is evident in *Ips confusus* transcripts (GIWS00000000), after our analysis developed on this project.

In other insects, it has been postulated that the existence of gene copies of α-amylase could be associated with an increase in their adaptive capacities to different food sources or to the occurrence of plant inhibitors [33,34]. Although the presence of inhibitors in the phloem pine trees has been documented, *Dendroctonus*-bark beetles feed only on phloem. Stable and functionally generalist enzymes may be more efficient in an environment heterogeneous such as the subcortical region of the pine tree, which is degraded and changed as colonization proceeds and the tree dies.

The phylogenetic analysis of AmyDr revealed the existence of a close relationship with the α-amylases of Curculionidae, and from these to those of Chrysomelidae, suggesting a common evolutionary history between both families of α-amylases. In addition, the presence of a short “flexible loop” with the absence of GHGA/GHG motif near the catalytic cleft in AmyDr and α-amylases from weevils and leaf beetles, suggests that this condition is conserved. Thus, like in other insect orders, two conditions exist in Coleoptera, namely one α-amylase group without motif and with a shorter “flexible loop” and the other group having a “flexible loop” with the presence of the GHGA/GHG motif [31]. While both conditions have widely been documented, there are no convincing functional explanations or complete phylogenetic patterns that could enable us to hypothesize and explore why both conditions have evolved.

On the other hand, in contrast to both chlorine-dependent (arginine-bearing α-amylases) and chlorine-independent (glutamine-bearing α-amylases) α-amylases, AmyDr has a lysine (LYS319 without peptide-signal) in the third residue of the binding site to chlorine ion. This mutation is shared with other scolytines species (e.g., *Dendroctonus*, *Ips*, *Hypotenemus*), weevils (e.g., *Anthonomus grandis*), chrysomelids (e.g., *Callosobruchus chinensis* and *Zabrotes subfasciatus*), and the two leaf beetle species analyzed (Figure S1), as well as with the bacteria *Alteromonas haloplanktis* [35]. This mutation favours the union of ions NO_3_^−^ and Cl^−^, which have been reported in other insect studies as weak activators. Our docking analyses showed that the binding of NO_3_^−^ to AmyDr (ARG182, ASN283, LYS319) is slightly stronger (binding energy −17.2 kJ mol^−1^ and hydrogen bonds with distances of ≤2.2 Å) than that of Cl^−^ (−16.74 kJ mol^−1^ and ≤2.1 Å), as previously demonstrated in marine bacterium *A. haloplanktis* [36].

Experimental results confirm the docking prediction because the activity of recombinant AmyDr is enhanced by the ions Ca^+2^, and NO_3_^−^, and slightly less by Cl^−^. These findings indicate that AmyDr is conformationally stable and maintains its activity either in the presence or absence of these last two ions. It is known that insect α-amylases require calcium ions for the structural integrity and stability, and are activated by nitrates and chlorine ions. However, to our knowledge, there are no reports in other insects of lysine-bearing α-amylases or α-amylases with this mutation, functioning both in the absence and presence of these ions. The functional flexibility of α-amylase in bark beetles, as well as some weevils and phytophagous insects, suggests that this feature could be a functional adaptation associated with their habitat and feeding. In the case of *Dendroctonus* bark beetles, this could be an adaptation to the changing subcortical environment where they develop and feed, which is apparently poor in calcium, nitrogen, and chlorine ions. These ions are acquired by trees through mineralization of the organic matter in the soil, where significant amounts of them are present [37]. However, the nitrogen in bark beetles’ gut is mainly the result of the activity of their symbionts [5,38], and calcium and chlorine are acquired mainly through feeding but are actively regulated in the insect’s body by their functional role in different physiological processes.

The observed expression of *AmyDr* gene in the head-pronotum in different developmental stages, suggests that starch hydrolysis starts in the oral cavity of *D. rhizophagus*. Whereas there is an obvious connection between starch hydrolysis and the nutrition process mainly in the midgut, amylolytic activity in the head-pronotum, salivary glands, and extraoral secretions have been frequently recorded in different insect species. Some authors have hypothesized that the lower amylolytic activity in the head-pronotum region, salivary glands, and extraoral secretions compared to the gut could be explained by the lack of optimal pH for the activity of α-amylases [23,39]. However, while this hypothesis may be applied to bark beetles, other factors, that might limit its activity, should be taken into consideration. For example, the high sclerotization of the anterior part of the bark beetles digestive system, which includes the oral cavity, pharynx, crop, and foregut, is structurally more associated to mechanic actions than digestive functions [40,41,42]; the presence of inhibitors in the phloem cells that are released when the insect crushes the phloem [34,43]; and the strong energy demand that the synthesis of terpenoids represents, which are present in the induced resin that is produced once the insect colonizes the tree as part of the tree defence system [44].

Lastly, the sharp decline in *AmyDr* gene expression levels at the pupa and pre-imago stages compared to pre-emerged adults and larvae, indicates that the activity of the digestive processes in the gut is minimum at those stages. In addition, the general pattern of *AmyDr* gene expression in this system suggests that the amylolytic activity is regulated through insect development. The different *AmyDr* gene expression levels observed among the developmental stages of *D. rhizophagus* can be explained by the processes of morphogenesis and organogenesis that occurs during the metamorphosis from larval-pupa, and pupa-pre-imago that limited food ingestion. On the other hand, we hypothesized that the regulation of amylolytic activity in the bark beetle may be associated with starch availability in the host tree. Several studies have reported that starch is accumulated in conifer needles, stem bark, and roots during spring [45], just when adults of this bark beetle are found in the tree roots waiting to emerge in early summer with the start of the rain season [46]. The growth and feeding of larval stages (I–IV) during summer and autumn matches with trees growth, when starch is hydrolyzed and its products moved to different parts of the tree to cover this energy cost. The fifth instar larvae finally migrate toward tree roots during early winter, where important starch reserves are stored. This hypothesis should be tested in further studies.

## 4. Materials and Methods

### 4.1. Insects

Naturally infested Arizona pines (*Pinus arizonica* Engelm) were collected at Mil Diez locality (23°48′28.12″ N 105°24′11.63″ W), Pueblo Nuevo municipality, Durango state, Mexico, from 2018 to 2019, and were transported immediately to the laboratory to get fifth-instar larvae (L5), pupa (PU), pre-imago (PI), and pre-emergent adult (AD) of *D. rhizophagus*. The gut was dorsally extracted and separated from the fat body and the Malpighian tubules; the head-pronotum and gut sections were separately placed into Eppendorf vials with 200 µL of Trizol^®^ (Life Technologies, Carlsbad, CA, USA), and macerated completely using a sterile pestle. Three biological replicates of each stage were processed. As negative control, we used eggs collected directly from the galleries with fine-tip forceps. Three egg groups (*n* = 150) were integrated and placed in Eppendorf vials in the field, and several consecutive washes were done with distilled water and PBS buffer, and finally, they were stored in 200 µL of Trizol^®^. In the laboratory, they were completely macerated using a sterile pestle. For all the samples, vials were topped with 1 mL of Trizol^®^ and stored at −80 °C for posterior RNA extraction and subsequent RT-qPCR.

### 4.2. DNA Cloning, Sequencing, and Full-Length Sequence Analyses

Total RNA was isolated from the whole adult body using the RiboPure™ RNA Purification kit (Life Technologies, Carlsbad, CA, USA) following the manufacturer’s instructions. The cDNA was synthesized from 2 µg of total RNA using the High-Capacity cDNA Reverse Transcription kit (Applied Biosystems, Carlsbad, CA, USA) as per manufacturer’s protocol, and stored at −20 °C. Forward primer F1 5′ TGGCGATCAGTTCTAGACCAA 3′ and reverse primer R1 5′ ATTGCAACGTGCTCTCATAA 3′ were designed according to UTRs of a *D. rhizophagus*’ putative α-amylase found in the transcriptome of this species [30]. This sequence was similar to a putative α-amylase found in the genome of *D. ponderosae* (XM_019912291.1) through Blastn, and to an α-amylase (AmyA, HQ417115) from the bark beetle *Ips typographus* reported by [25].

The PCR reaction was performed using Dream Taq Polymerase (Invitrogen, CA, USA) and the following conditions: 94 °C for 3 min, followed by 35 cycles of denaturation at 94 °C for 30 s, 57.5 °C annealing for 30 s, and extension at 72 °C for 2 min, with a final extension step at 72 °C for 20 min. PCR products were cloned into pGEM T Easy Vector (Promega, Madison, WI, USA) following the manufacturer’s instructions. A total of 30 clones with insert were sequenced in both senses at Macrogen, Inc. (Geumcheon-gu, Seoul, Korea).

The sequence obtained by PCR was confirmed by BlastX. To search different isoforms, the putative α-amylase sequence was mapped against all transcripts of adults and pupae transcriptomes from *D. rhizophagus* [30] using GMAP software, v. 2017-11-15 [47] with a kmer of 12. An α-amylase encoding gene (*AmyDr*) was deposited in the GenBank (accession number MN782509). The deduced amino acid sequence was obtained with the ExPasy Translate Tool (http://www.expasy.org/tools/dna) and the Protein isoelectric point calculator (http://isoelectric.org) was used to predict the physicochemical characteristics of the enzyme, including molecular mass (kDa) and isoelectric point (pI). Moreover, subcellular localization and signal peptide predictions were realized with the ProtComp-AN software, v.9.0 and SignalP 5.0 server (http://www.cbs.dtu.dk/services/SignalP). The NetNGlyc v.1.0 (http://www.cbs.dtu.dk/services/NetNGlyc) and NetOGlyc v.4.0 (http://www.cbs.dtu.dk/services/NetOGlyc) servers were used to predict potential N and O glycosylation sites.

### 4.3. Phylogenetic Analysis

Multiple protein alignment with reference coleopteran sequences was performed with Muscle software, v.3.8.31 [48] and a phylogenetic inference analysis by maximum likelihood was carried out in PhyML 3.0 server (http://www.atgc-montpellier.fr/phyml/). The more appropriate model of protein evolution was determined using the Akaike information criteria (AIC = 25857.30, − lnL = −12834.65) and Bayesian information criteria (BIC = 26253.66, − lnL = −12834.65) [49]. Both criteria supported the model LG + G + I + F [50], and the support of nodes was evaluated after 1000 bootstrap pseudoreplicates. The α-amylase sequence of *Spodoptera frugiperda* (AAO13754.1) was used as outgroup.

### 4.4. Molecular Modelling and Docking

The 3D structure of the α-amylase was obtained by homology modelling with Modeller software, v.9.16 [51], using as a template the α-amylase crystalized structure obtained by the X-Ray of *Tenebrio molitor* (PDB ID: 1TMQ, 1JAE), which is the only structural model of α-amylases in insects elucidated by X-Ray [52]. The best model was selected and validated in the Structural Analysis and Verification Server (http://servicesn.mbi.ucla.edu/SAVES) using Verify3D [53], Errat [54], Procheck [55], and Whatcheck [56]. The Ramachandran plot was generated [57] in the RAMPAGE server (http://mordred.bioc.cam.ac.uk/~rapper/rampage.php). To identify active and binding sites with calcium and chlorine ions, a multiple alignment without signal peptides of AmyDr sequence from *D. rhizophagus* and several α-amylases from coleopterans and lepidopterans was performed in ESPript software, v.3.0 [58]. The 3D structure of the α-amylase of this bark beetle was analyzed using the PyMol™ software, v.2.2.0 [59].

Predicted interactions between amylopectin and amylose with ions were evaluated using docking analyses. Ligands were drawn in ChemDraw software, v.15.0 (http://www.cambridgesoft.com) and were geometrically optimized with the Gaussian software, v.5.0.9 [60] using AM1 and DFT (B3LYP/6-31G(d,p)) levels. The output files were converted to .pdb files, and the ligand files were viewed using the AutoDockTools (ADT software), v.4.2 [61]. To perform docking analyses, the Kollman charges for all atoms were computed prior to the addition of polar hydrogens to the α-amylase, and torsion angles in the small flexible ligands were included in the Monte Carlo algorithm. The protein exploration and definition of the binding site were prepared using a GRID-based procedure [62]. All docking simulations employed the hybrid Lamarckian genetic algorithm with an initial population of 100 randomly placed individuals and 1 × 10^7^ energy evaluations. The interactions between the ligands and the α-amylase were visualized using ADT software, v.4.2, and the figures were created using the PyMol™ software, v.2.2.0.

### 4.5. Amylolytic Activity and Peptide Identification

Ten adult beetles of *D. rhizophagus* (1:1 sex ratio) were placed in holes drilled (0.5 cm) into the fresh stumps of Arizona pines (40 cm high and ~15 cm diameter) and secured with steel mesh and staples. The beetles were fed for 12 h at room temperature in dark conditions. Then, the head-pronotum was removed directly from the insects, and the guts were dorsally extracted after the elytra and wings. The head-pronotum and gut from insects were separately homogenized in 100 µL 1 mM phenylmethylsulfonyl fluoride (PMSF) and centrifuged at 10,000× *g* for 15 min at 4 °C. The pellets of each sample were measured, normalized, and loaded by duplicate with the sample buffer (0.05 M Tris HCl, pH 7.0; 20% glycerol, 0.05% Triton X-100; 0.01% bromophenol blue) and incubated at 37 °C for 10 min. The samples were loaded twice into an 8% polyacrylamide gel copolymerized with 1% (w/v) of starch (Sigma-Aldrich, St. Louis, MO, USA) using a Mini PROTEAN II system with a running buffer free of SDS at pH 8.3. Native-PAGE was conducted at 4 °C for 4 h at 60 V. After electrophoresis, the gel was rinsed with distilled water for 15 min, and the zymogram assay was performed in 50 mM citrate buffer, pH 5.0, for 1 h at 40 °C.

The gel was cut into two longitudinal sections. The first section was stained with Lugol’s solution for 5 min and incubated in distilled water with shaking to visualize the amylolytic activity. The second section was not stained but was matched with the first section once activity was visualized. To determinate the presence of α-amylase peptides from the head-pronotum and gut samples using MALDI-TOF/TOF, the lane was divided into four parts (S1–S4). Due to amylolytic activity displayed in the section S3 in both samples, it was divided into two subsections (a, b) for the gut sample and (a’, b’) for the head-pronotum sample. These sections were cut and digested with trypsin to identify peptides according to Shevchenko et al. [63]. The resulting peptides were concentrated in an approximate volume of 10 μL. Thereafter, 9 μL was desalted using C18 Zip-Tips (Millipore, Burlington, MA, USA). ChromXP Trap Column C18-CL precolumn (Eksigent, Dublin, CA, USA); 350 μm X 0.5 mm, 120 Å pore size, 3 μm particle size and desalted with 0.1% trifluoroacetic acid (TFA) in H_2_O at a flow rate of 5 μL min^-1^ during 10 min. Then, peptides were loaded and separated on a 3C18-CL-120 column (Eksigent, Dublin, CA, USA); 75 μm X 150 mm, 120 Å pore size, 3 μm particle size, in an HPLC Ekspert nanoLC 425 (Eksigent, Dublin, CA, USA) using as mobile phase A, 0.1% TFA in H_2_O, and mobile phase B 0.1% TFA in acetonitrile (ACN) under the following linear gradient: 0-3 min 10% B, 60 min 60% B, 61–64 min 90% B, 65 to 90 min 10% B at a flow rate of 250 µL min^−1^. Eluted fractions were automatically mixed with a solution of 2 mg mL^−1^ of α-cyano-4-hydroxycinnamic acid in 0.1% TFA and 50% ACN as a matrix, spotted in an Opti-TOF plate of 384 spots using a MALDI Ekspot (Eksigent, Dublin, CA, USA) with a spotting velocity of 20 s per spot at a matrix flow rate of 1.6 μL min^−1^. The spots generated were analyzed by a MALDI-TOF/TOF 4800 Plus mass spectrometer (ABSciex, Framingham, MA, USA). Each MS Spectrum was acquired by an accumulation of 1000 shots in a mass range of 850–4000 Th with a laser intensity of 3800. The 100 more intense ions with a minimum signal-noise of 20 were programmed to fragmenting. The MS/MS spectrums were obtained by the fragmentation of selected precursor ions using collision-induced dissociation and acquired by 3000 shots with a laser intensity of 4300. Generated MS/MS spectrums were compared against the Swiss-Prot database by the Protein Pilot software, v.2.0.1, using the Paragon algorithm. Search parameters were: Carbamidomethylated cysteine, trypsin as a cut enzyme, all the biological modifications and amino acid substitution set by the algorithm; as well as phosphorylation emphasis, and Gel-based ID as special factors. The detection threshold was considered in 1.3 to acquire 95% of confidence. Also, the identified proteins observed a local false discovery rate of ≤ 5%. In a bid to minimize redundancy, identified proteins were grouped by the ProGroup algorithm in Protein Pilot software, v.2.0.1. Analyses were carried out by the “Unidad de Genómica, Proteómica y Metabolómica” at LaNSE, CINVESTAV, CDMX, México.

### 4.6. RT-qPCR

The relative expression of the α-amylase was evaluated in the different developmental stages of the *D. rhizophagus* life cycle (L5, PU, PI, and AD) and the different tissues (head-pronotum and gut). Total RNA from the samples was isolated using the RiboPure™ RNA Purification kit (Life Technologies, Carlsbad, CA, USA). The RNA concentration and purity (A_260_/A_280_ ratio) were determined using an ND-2000 spectrophotometer (Thermo Fisher Scientific, Waltham, MA, USA) and its integrity was checked in 1% agarose gel electrophoresis. The cDNA synthesis was done using the High-Capacity cDNA Reverse Transcription Kit (Applied Biosystems, Carlsbad, CA, USA) from 2 μg total RNA in a final reaction volume of 20 μL, as per manufacturer’s protocol. TaqMan^®^ probes and primers were designed by Applied Biosystems (Table S1). The TaqMan^®^ hydrolysis probes were labeled at the 5′ end with the reporter dye 6-carboxyfluorescein (FAM) and joined at the 3’ end to a non-fluorescent quencher molecule group MGB (Minor Groove Binder). The reactions were carried out with a Step One^®^ TM Real-Time PCR System (Applied Biosystems, Carlsbad, CA, USA) in a final volume of 20 μL: 20X primer and probes with a final concentration of 900 nM of each primer, 250 nM of the TaqMan probe, TaqMan^®^ Universal Master Mix II (Applied Biosystems, Carlsbad, CA, USA) and 5 μL of 100-fold dilution of cDNA. All samples were placed in Fast Optical 48-well reaction plates MicroAmp^®^™ (Thermo Fisher Scientific, Carlsbad, CA, USA). Standard manufacturer’s amplification conditions were used: 50 °C for 2 min, 95 °C for 10 min, 40 cycles at 95 °C for 15 s, and 60 °C for 60 s. PCR contamination was undetected in the no template control NTCs (unintended amplification products) and three technical replicates were performed for each biological replicate from each development state. A set of ten guts or ten head-pronotum were used in each of the replicas.

Glyceraldehyde 3-phosphate dehydrogenase (*GAPDH*; MN782508) was used as a reference gene since it showed a stable expression according to pupa and adult transcriptomes of *D. rhizophagus* [30]. The RT-qPCR efficiency and validation for the reference gene were assessed using a linear regression analysis with average values obtained in three replicates of the quantification cycles (Ct) with five cDNA dilutions (an initial 1:20 dilution and four subsequent 1:5 dilutions). The PCR efficiency for *GAPDH* and *Amy* genes were estimated with the relation: efficiency = (10^−1/slope^−1)100, where the slope values were −3.58 and −3.52 respectively, and R^2^ value of 0.99. To evaluate the specificity, all amplicons were visualized in 1% agarose gel electrophoresis. All experimental procedures were performed according to the Minimum Information for Publication of Quantitative Real-Time PCR Experiments (MIQE) guidelines (Supplementary Table S2) [64].

### 4.7. Statistical Analysis

The relative expression values of the α-amylase gene were determined using the 2^−ΔΔCt^ method [65], where the Ct values of the egg stage and the *GAPDH* gene were used for data normalization. To evaluate significant differences in the expression level of the α-amylase gene, a one-way ANOVA with Welch’s *F* and Tukey test were performed for the two different tissues (head-pronotum and gut) and different developmental stages (fifth-instar larvae, pupa, pre-imago, and pre-emergent adult). Statistical tests were performed with Past software, v.3.26 [66]. Values of *p* < 0.05 were considered significant.

### 4.8. Expression of Recombinant AmyDr

Plasmid with recombinant *AmyDr* was recovered from DH5α cells with the construction pGEM_*AmyDr*, using the GenElute TM Plasmid Midiprep kit (Sigma-Aldrich Corp., St. Louis, MO, USA) following manufacturer’s protocol. The *AmyDr* open reading frame (ORF) was PCR-amplified with primers F1_DR1_SPIF (5′GTCGACTGGATCCGGATGCAGCTAGGAATTCTG3′) and R1_DR1_SPIF (5′ATCTCGAGTGCGGCCTTATAATTTTGCATTTACATG3′) using a Q5 High-Fidelity DNA Polymerase (New England BioLabs, Inc., Ipswigh, MA, USA) following manufacturer’s protocol. The amplification product was cloned into pENTR4 using In-Fusion HD EcoDry (Takara Bio, inc., Mountain View, CA, USA) and transformed into Stellar™ Competent Cells (*E*. *coli* HST08) (Takara Bio, inc., Mountain View, CA, USA) following the manufacter’s instructions. The recombinant plasmid was confirmed by sequencing prior recombination into the linearized BaculoDirect™ C-term vector (Invitrogen, Carlsbad, CA, USA) by LR recombination with LR Clonase™ II (Invitrogen, Carlsbad, CA, USA). *Sf9* cells were infected with the recombinant Bac_*AmyDr* vector to obtain a high-titer P3 viral stock by successive amplifications of P1 and P2 stocks following manufacturer’s protocol. 

Recombinant *AmyDr* P3 viral stock (500 μL), previously produced, was used to infect 1 × 10^6^ *Sf9* cells mL^−1^ in 30 mL of Sf900 II serum-free media (Gibco, Grand Island, NY, USA) suspension cultures for 72 h at 27 °C. Cells expressing recombinant *AmyDr* were harvested with Cell Lysis Buffer (100 mM sodium phosphate, pH 7.6, 20% (vol/vol) glycerol, and 1.1 mM EDTA) supplemented with 75 μL of phenylmethylsulfonyl fluoride (0.1 M PMSF), 15 μL of protease inhibitor cocktail (PIC) (Sigma-Aldrich Corp., Milwaukee, WI, USA), and 30 μL of dithiothreitol (0.1 M DTT), and then centrifuged at 3000× *g* at 4 °C for 10 min. The supernatant was isolated and tested for functional AmyDr. As negative control of amylases expression, 30 mL of 1 × 10^6^ *Sf9* cells mL^−1^ in Sf-900 II SFM suspension culture without viral stock and processed as mentioned before was used.

### 4.9. Preparation of Amylase Crude Extract

The total protein from AmyDr crude extract and the negative control from *Sf*9 cells without viral stock were precipitated overnight at 4 °C and 80% (NH_4_)_2_SO_4_ saturation. The pellet was recovered by centrifugation at 8800× *g* at 4 °C for 20 min, suspended in 5 mL of 50 mM, pH 5.0 citrate buffer, and dialyzed at 4 °C for 12 h in a cellulose membrane (Spectrum, Zarauz, Guipúzcoa, Spain) using the same buffer. Proteins obtained were isolated in 10% SDS-PAGE.

### 4.10. Protein Electrophoresis

Protein analyses were performed in 10% SDS-PAGE using a Mini PROTEAN II system. Proteins in the gel were visualized using Coomassie Brilliant Blue R-250 and recorded with a MiniBIS Pro documentation system (DNR Bio-Imaging Systems Ltd., Jerusalem, Israel). Protein molecular weight (MW) was estimated using as reference broad-range molecular weight protein standards (Thermo Fisher Scientific, Waltham, MA, USA).

### 4.11. Enzyme Assay

The amylolytic activity of AmyDr was determined on 1% starch agar plates (10 g L^−1^ starch, 23 g L^−1^ agar dissolved in deionized water), where 50 μL of amylase crude extract were added in a 5 mm hole, previously made with a Pasteur pipette on starch agar plates. These were incubated at 37 °C for 12h and stained with Lugol’s solution to document the enzyme activity around the hole.

### 4.12. Effect of Metal and Non-Metal Anions on Enzyme Activity

To evaluate the effect of metal ions, such as Ca^2+^ and Cl^−^, and non-metal such as NO_3_^−^ on the enzymatic activity of AmyDr, samples of the crude extract were incubated in CaCl_2_, NH_4_Cl, Ca(NO_3_)_2,_ and KNO_3_ at two final concentrations of 1 and 5 mM each. Likewise, as described before, the reaction mixture was added in a 5-mm hole on the starch agar plates, incubated at 37 °C for 12 h, and stained with Lugol’s solution to document the enzyme activity around the hole. Amylolytic activity was compared to a control plate with only metal and non-metal ions, and a control plate with *Sf*9 cells including metal and non-metal ions, which were the negative control of this assay.

## Supplementary Materials

Supplementary materials can be found at https://www.mdpi.com/1422-0067/22/1/36/s1. Figure S1. AmyDr sequence and multiple sequence alignment, assignment of the secondary structural elements; Table S1. TaqMan probes; Table S2. MIQUE checklist.

## Abbreviations

L5	Fifth-instar larvae
PU	Pupa
PI	Pre-imago
AD RT-qPCR GAPDH AIC BIC PMSF PIC DTT SDS-PAGE TMA TFA NTCs	Pre-emergent adult Quantitative real-time Polymerase Chain Reaction Glyceraldehyde 3-phosphate dehydrogenase Akaike information criteria Bayesian information criteria Phenyl-methyl-sulfonyl-fluoride Protease inhibitor cocktail Dithiothreitol Sodium Dodecyl Sulfate Polyacrylamide Gel Electrophoresis *Tenebrio molitor* amylase Trifluoroacetic acid Unintended amplification products
