# Supplementary material for: The Bark Beetle Dendroctonus rhizophagus (Curculionidae: Scolytinae) Has Digestive Capacity to Degrade Complex Substrates: Functional Characterization and Heterologous Expression of an α-Amylase
Source: Int J Mol Sci. 2020 Dec 22;22(1):36. doi: 10.3390/ijms22010036 (PMC7792934; doi:10.3390/ijms22010036)
Supplement: Supplementary file 1 [file ijms-22-00036-s001.zip › Figure S1.pdf]

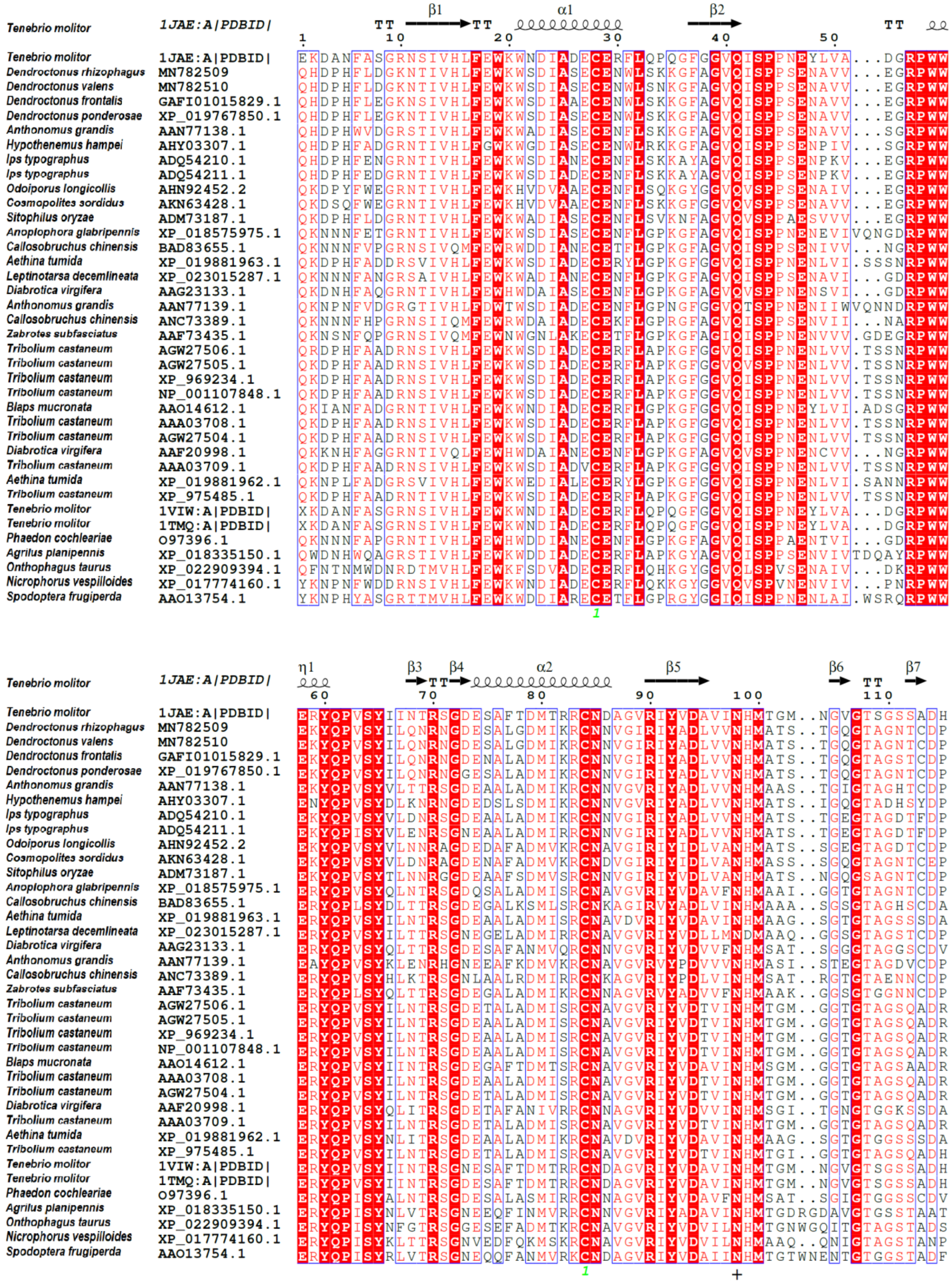

Tenebrio molitor 1JAE:A|PDBID|
β1 α1 β2 TT
1 10 20 30 40 50
Tenebrio molitor 1JAE:A|PDBID|
Dendroctonus rhizophagus MN782509
Dendroctonus valens MN782510
Dendroctonus frontalis GAFI01015829.1
Dendroctonus ponderosae XP_019767850.1
Anthonomus grandis AAN77138.1
Hypothenemus hampei AHY03307.1
Ips typographus ADQ54210.1
Ips typographus ADQ54211.1
Odoiporus longicollis AHN92452.2
Cosmopolites sordidus AKN63428.1
Sitophilus oryzae ADM73187.1
Anoplophora glabripennis XP_018575975.1
Callosobruchus chinensis BAD83655.1
Aethina tumida XP_019881963.1
Leptinotarsa decemlineata XP_023015287.1
Diabrotica virgifera AAG23133.1
Anthonomus grandis AAN77139.1
Callosobruchus chinensis ANC73389.1
Zabrotes subfasciatus AAF73435.1
Tribolium castaneum AGW27506.1
Tribolium castaneum AGW27505.1
Tribolium castaneum XP_969234.1
Tribolium castaneum NP_001107848.1
Blaps mucronata AAO14612.1
Tribolium castaneum AAA03708.1
Tribolium castaneum AGW27504.1
Diabrotica virgifera AAF20998.1
Tribolium castaneum AAA03709.1
Aethina tumida XP_019881962.1
Tribolium castaneum XP_975485.1
Tenebrio molitor 1VIW:A|PDBID|
Tenebrio molitor 1TMQ:A|PDBID|
Phaedon cochleariae O97396.1
Agrilus planipennis XP_018335150.1
Onthophagus taurus XP_022909394.1
Nicrophorus vespilloides XP_017774160.1
Spodoptera frugiperda AAO13754.1
1
η1 β3 β4 α2 β5 β6 β7
60 70 80 90 100 110
+

```
Tenebrio molitor          1JAE:A|PDBID|        η2                    α3                  α4
                                          120       130       140          150       160       170

Tenebrio molitor          1JAE:A|PDBID|     DGMNYPAVPYGSGDFH.SPCEVNNYQ...DADNVRNCELVGLRDLNQGSDYVRGVLIDYM
Dendroctonus rhizophagus  MN782509          SSKSYPAVSYSEENFH.TSCDI.DYN...DAASIRNCELSGLKDLDQSQDYVRGKIIEYM
Dendroctonus valens       MN782510          SSKSYPAVSYSEENFH.TSCDI.DYN...DAASIRNCELSGLKDLDQSQDYVRGKIVEYM
Dendroctonus frontalis    GAFI01015829.1    SSKSYPAVSYSEENFH.TSCDI.DYN...DASSIRNCELSGLKDLDQSQDYVRGKIVEYI
Dendroctonus ponderosae   XP_019767850.1    SSKSYPAVSYSEENFH.TSCDI.DYN...DAASIRNCELSGLKDLDQSQDYVRGKIVEYM
Anthonomus grandis        AAN77138.1        GSKSYPAVSYSSENFH.ATCDI.NYN...DAASIRNCELSGLKDLDQSQDYVRGKIIEYM
Hypothenemus hampei       AHY03307.1        GSKSYPAVPYSNENFH.ASCDI.DYN...DAASIRNCELSGLKDLDQSQDYVRGKIVDYM
Ips typographus           ADQ54210.1        DTKSYPTVPYTSENFH.TSCEI.DYT...DAASIRNCELSGLKDLDQSQEYVRGKIIDYI
Ips typographus           ADQ54211.1        DTKSYPAVPYSSENFH.PSCDI.DYS...DASSIRNCELVALKDLDQSQEYVRGKIIDYI
Odoiporus longicollis     AHN92452.2        GSKSYPAVSYSSENFH.PSCDI.DYN...DAASIRNCELSGLKDLDQSQDYVRGKIIDYL
Cosmopolites sordidus     AKN63428.1        GSKSYPAVSYSSENFH.PTCDI.NYN...DAASIRNCELSGLKDLDQSQDYVRGKIIDYL
Sitophilus oryzae         ADM73187.1        SSKSYPAVSYTSENFH.TSCDI.DYT...DSSSIRNCELTGLKDLDQSQDYVRGKIEEYM
Anoplophora glabripennis  XP_018575975.1    DSKSYPGVPYGSDNFH.TTCTVNNYN...DAENVRNCELVGLKDLDQSQDYVRQKIVAFL
Callosobruchus chinensis  BAD83655.1        GSRSYPTVPYGGADFH.KSCPINNYN...DPAEVRNCELVGLPDLDQGRQYVKDKIVEYM
Aethina tumida            XP_019881963.1    DNKKFPGVSYGSDDFH.ASCRIDNYQ...DAENVRNCELEGLKDLDQSHAYVRQKIVEYM
Leptinotarsa decemlineata XP_023015287.1    GSKYFPAVPYNSDDFH.PTCTVNNYN...DAANVRNCELVGLPDLDQSKDGVRQKLVAYI
Diabrotica virgifera      AAG23133.1        GSLSYPSVPFGSNDFH.SKCDVNNYQ...DANNIRNCWLSGLPDLDQSHDYVRQKIVEYL
Anthonomus grandis        AAN77139.1        TNRDYPAVPYTIEHFH.PTCGM.DYN...SPSAIRNCELSGLPDLNQTEDYVREKIKDYM
Callosobruchus chinensis  ANC73389.1        GSRSYPAVPYGGGDFH.KSCPLNNYG...DPAEVRNCELVGLPDLDQSKQYVRDKIVEYM
Zabrotes subfasciatus     AAF73435.1        SKKSYPAVPYGPDDFH.PDCMINNYQ...DVNNVRNCQLVGLPDLDQSKQYVRDKIVGYL
Tribolium castaneum       AGW27506.1        DGKNYPAVPYGSGDFH.DSCTVNNYQ...DASNVRNCELVGLADLNQGSDYVRSKIIEYM
Tribolium castaneum       AGW27505.1        DGKNYPAVPYGSGDFH.DSCTVNNYQ...DASNVRNCELVGLADLNQGSDYVRSKIIEYM
Tribolium castaneum       XP_969234.1       DGKNYPAVPYGSGDFH.DSCTVNNYQ...DASNVRNCELVGLADLNQGSDYVRSKIIEYM
Tribolium castaneum       NP_001107848.1    DGKNYPAVPYGSGDFH.DSCTVNNYQ...DASNVRNCELVGLADLNQGSDYVRSKIIEYM
Blaps mucronata           AAO14612.1        AGKNYPGVPYGSGDFH.DSCAINDYQ...DTNNVRNCELEGLADLDQGSEYVRGKIIEYM
Tribolium castaneum       AAA03708.1        DGKNYPAVPYGSGDFH.DSCTVNNYQ...DASNVRNCELVGLADLNQGSDYVRSKIIEYM
Tribolium castaneum       AGW27504.1        DGKNYPAVPYGSGDFH.DSCTVNNYQ...DASNVRNCELVGLADLNQGSDYVRSKIIEYM
Diabrotica virgifera      AAF20998.1        GSLSYPGVPFGPNHFH.SRCDINNYQ...ESHNIRNCALTGLPDLDQSHEHVRKKIVEYL
Tribolium castaneum       AAA03709.1        DGKNYPAVPYGSGDFH.DSCTVNNYQ...DASNVRNCELVGLADLNQGSDYVRSKIIEYM
Aethina tumida            XP_019881962.1    GNKQFPGIPYGPGDFH.DSCAINNYQ...DASNVRNCELEGLKDLDQSHDYVRQKIVEYM
Tribolium castaneum       XP_975485.1       DGKNYPAVPYGPDDFH.EFCTINNYQ...DASNVRNCELEGLIDLNQSLDHVRSKIIEFM
Tenebrio molitor          1VIW:A|PDBID|     DGMNYPAVPYGSGDFH.SPCEVNNYQ...DADNVRNCELVGLRDLNAGSDYVRGVLIDYM
Tenebrio molitor          1TMQ:A|PDBID|     DGMNYPAVPYGSGDFH.SPCEVNNYQ...DADNVRNCELVGLRDLNQGSDYVRGVLIDYM
Phaedon cochleariae       O97396.1          EPSASPAVPYGSGDFH.GRCTSNNYQ...DPNNIRNCWLSGLPDLDQSKDYVRDKILEYL
Agrilus planipennis       XP_018335150.1    SSEEYPAVPYHAEHFH.PSCSVNNYN...DANNVRNCELSGLKDLDQSNSYVAGKIIDYM
Onthophagus taurus        XP_022909394.1    AIKSYPAVPYSAPDFN.SDCAITDYN...NAENVRNCELVGLRDLHQGSEYVRGKMVDFL
Nicrophorus vespilloides  XP_017774160.1    GSKEYPAVPYGPNDFH.PDCSINDYN...NATEVRDCELVGLKDLNQGTNYVRGKLVEFL
Spodoptera frugiperda     AAO13754.1        GNWGYPGVPYGRNDFNWPHCVIQGHDYGCCADRVRNCELSGLKDLNQGNEYVRQQIVNYM
                                                               2                +  2     +
```

```
Tenebrio molitor          1JAE:A|PDBID|          β8     η3      α5          η4  TT    β9
                                                180       190       200       210       220       230

Tenebrio molitor          1JAE:A|PDBID|     NHMIDLGVAGFRVDAAKHMSPGDLSVIFSGLKNLNTDYGFADGARPFIYQEVIDLGGEAI
Dendroctonus rhizophagus  MN782509          NHLISLGVAGFRVDAAKHMWPADLSEMFGSVNDLNTEF.FSEGSKPLFYQEVIDTGSDPI
Dendroctonus valens       MN782510          NHLISLGVAGFRVDAAKHMWPADLSEMFGSVNDLNTEF.FSEGSKALFYQEVIDTGSDPI
Dendroctonus frontalis    GAFI01015829.1    NHLISLGVAGFRVDAAKHMWPADLAEIFGRANDLSSEF.FSEGSKPLFYQEVIDTGTDPI
Dendroctonus ponderosae   XP_019767850.1    NHLISLGVAGFRVDAAKHMWPADLSEMFGSVNDLNTEF.FSEGSKPLFYQEVIDTGSDPI
Anthonomus grandis        AAN77138.1        NHLISLGVAGFRVDAAKHMWPADLSAIFGSVNDLNTDF.FPSGSRAMYYQEVIDTGSDPV
Hypothenemus hampei       AHY03307.1        NHLVSLGVAGFRVDAAKHMWPADLAAIFGSVNDLNTDF.FPSGSRAYIYQEVIDTGSDPI
Ips typographus           ADQ54210.1        NHLISLGVAGFRVDAAKHMWPEDLSVIFSSVNELNTEY.FPSGSKPLFYQEVIDTGTDPI
Ips typographus           ADQ54211.1        NHLISLGVAGFRVDAAKHMWPEDLSVIFSRVNELNTEY.FPSGSKPLFYQEVIDTGSDPV
Odoiporus longicollis     AHN92452.2        NHLVDLGVAGFRVDAAKHMWPADLEAIFGSVNDLNTDQGFDSGSRPFIFQEVIDTGTDPV
Cosmopolites sordidus     AKN63428.1        NHLVDLGVAGFRVDAAKHMWPADLEAIFGSVKDLNTDQGFPSGARAFIFQEVIDTGSDPV
Sitophilus oryzae         ADM73187.1        NHLISLGVAGFRVDAAKHMWPADLQAIFGSLNDLSTDHGFASGARAFIFQEVIDTSTDPV
Anoplophora glabripennis  XP_018575975.1    DQCVDLGVAGFRVDAAKHMWPADLQAIYGSVKDLSTDHGFASGTRPFFFQEVIDLGGEAI
Callosobruchus chinensis  BAD83655.1        NHLVDLGIAGFRVDAAKHIWPADLQAMYASVKNLNTEF.FPENSRPFYYQEVIDFGGDAI
Aethina tumida            XP_019881963.1    NHLIDLGVAGFRVDAAKHMWPADLEAIYGQLED..TQF....GGKPFIYQEVIDLGGEAI
Leptinotarsa decemlineata XP_023015287.1    DHLIDLGVAGFRVDAAKHMWPADLAAIYGMAKNLNTDHGFAEGSRPFFFQEVIDLGGEAI
Diabrotica virgifera      AAG23133.1        NHLVDLGVAGFRVDAAKHMWPADLEAIYGSVKDLT...GSGLSGRPFIYQEVIDLGGEAV
Anthonomus grandis        AAN77139.1        NHLIEIGVAGFRIDAAKHMWPEDLKIIYGSLNDLNTDY.FSAGSRPFFYQEVIDYGNDVV
Callosobruchus chinensis  ANC73389.1        NHLVDLGVAGFRVDAAKHIWPADLEAIYGRVKNLNTEF.FPANSRPFYYQEVVDFGGDAV
Zabrotes subfasciatus     AAF73435.1        NHLVDLGIAGFRVDAAKHMWPADLSAIYGSVKNLNSAY.FPGGSRPLFYQEVIDYGTEPI
Tribolium castaneum       AGW27506.1        NHLVDLGVAGFRVDAAKHMWPADLEAIYGSLKNLNTDHGFLDGQKPFIFQEVIDLGGEAI
Tribolium castaneum       AGW27505.1        NHLVDLGVAGFRVDAAKHMWPADLEAIYGSLKNLNTDHGFLDGQKPFIFQEVIDLGGEAI
Tribolium castaneum       XP_969234.1       NHLVDLGVAGFRVDAAKHMWPADLEAIYGSLKNLNTDHGFPDGQKPFIFQEVIDLGGEAI
Tribolium castaneum       NP_001107848.1    NHLVDLGVAGFRVDAAKHMWPADLEAIYASLKNLNTDHGFLDGQKPFIFQEVIDLGGEAI
Blaps mucronata           AAO14612.1        NHMVDLGVAGFRVDAAKHMWPADLEVIYGSLKNLNSDYGFSGSERPFIFQEVIDLGGEPI
Tribolium castaneum       AAA03708.1        NHLVDLGVAGFRVDAAKHMWPADLEAIYGSLKNLNTDHGFLLGQKPFIFQEVIDLGGEAI
Tribolium castaneum       AGW27504.1        NHLVDLGVAGFRVDAAKHMWPADLEAINGSLKNLNTDHGFLDGQKPFIFQEVIDLGGEAI
Diabrotica virgifera      AAF20998.1        NHLVDLGVAGFRVDAAKHMWPADLKAIYDSVKDLTGN...GLSGRPFVYQEVIDLGGEAV
Tribolium castaneum       AAA03709.1        NHLVDLGVAGFRVDAAKHMWPADLEAIYGSLKNLNTDHGFLDGQKPFIFQEVIDLGGEAI
Aethina tumida            XP_019881962.1    NHLVDLGVAGFRVDAAKHMWPADLEAIYGQLKD..TQF....GGKPFIYQEVIDLGGEPI
Tribolium castaneum       XP_975485.1       NHLVDLGVAGFRVDAAKHMWPADLEAIYGSLKYLNTNHGFLDGQKPFIFQEVVDFGGEAV
Tenebrio molitor          1VIW:A|PDBID|     NHMIDLGVAGFRVDAAKHMSPGDLSVIFDGLKNLNTDYGFADGARPFIYQEVIDLGGEAI
Tenebrio molitor          1TMQ:A|PDBID|     NHMIDLGVAGFRVDAAKHMSPGDLSVIFSGLKNLNTDYGFADGARPFIYQEVIDLGGEAI
Phaedon cochleariae       O97396.1          NHLVDLGVAGFRVDAAKHMWPADLQVIYGRVKDLNTDHGFSQGSRPFFYQEVIDLGGEGV
Agrilus planipennis       XP_018335150.1    NKLISYGVAGFRIDAAKHMTPSDLKVIYDSLNDLNTEYGFPANSKPFIYQEVIDLGGEAV
Onthophagus taurus        XP_022909394.1    NKLVNLGVAGFRIDAAKHMWPGDLEVILGRVNNLNTDYGFPSGARPYVYQEVIDLGGEGI
Nicrophorus vespilloides  XP_017774160.1    NNLIDLGVGGFRVDAAKHMWPEDLKVIYNQVKNLSVNYGFAPGSRPYIYQEVIDLGGEGI
Spodoptera frugiperda     AAO13754.1        NHLINLGVAGFRIDAGKHMWPGDLRVIYDRVHNLNTAHGFPSGARPYIYQEVIDLGGEVI
                                                       - *     +                              *
```

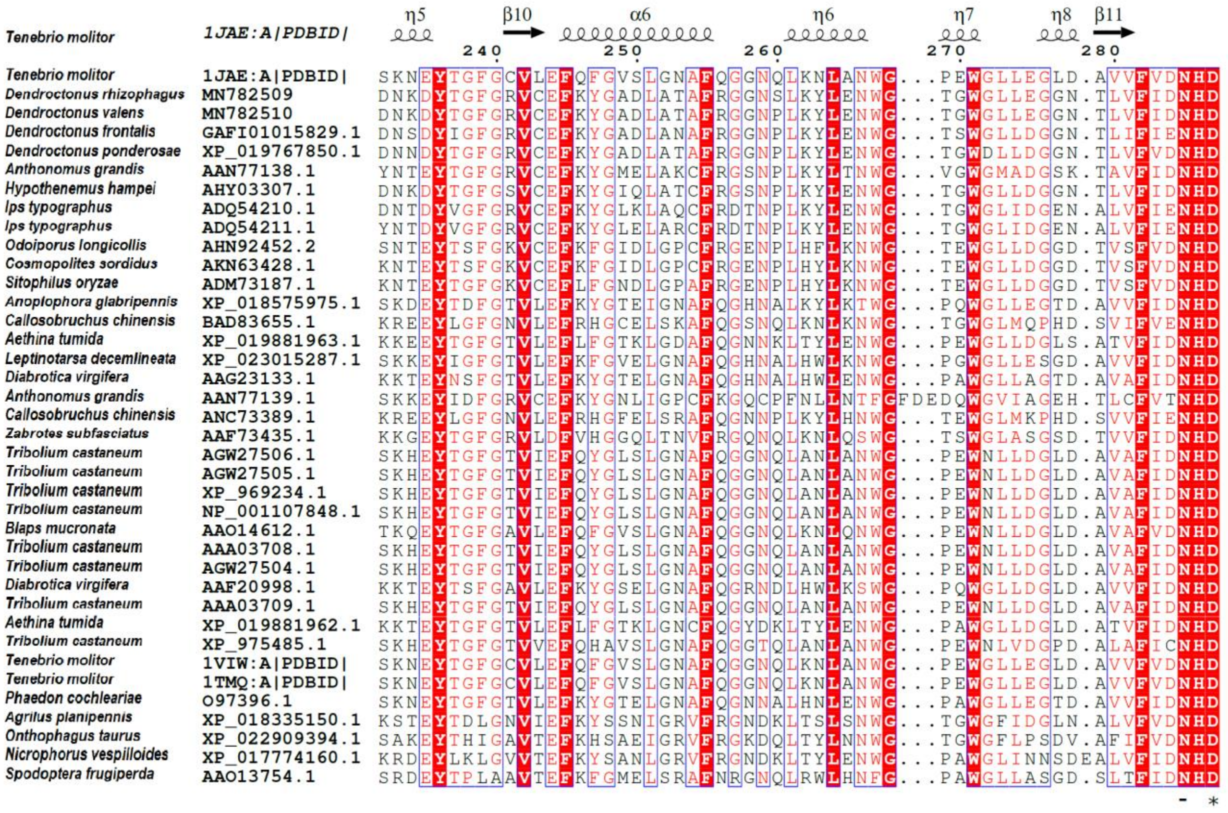

Tenebrio molitor 1JAE:A|PDBID|
η5 β10 α6 η6 η7 η8 β11
240 250 260 270 280
Tenebrio molitor 1JAE:A|PDBID|
Dendroctonus rhizophagus MN782509
Dendroctonus valens MN782510
Dendroctonus frontalis GAFI01015829.1
Dendroctonus ponderosae XP_019767850.1
Anthonomus grandis AAN77138.1
Hypothenemus hampei AHY03307.1
Ips typographus ADQ54210.1
Ips typographus ADQ54211.1
Odoiporus longicollis AHN92452.2
Cosmopolites sordidus AKN63428.1
Sitophilus oryzae ADM73187.1
Anoplophora glabripennis XP_018575975.1
Callosobruchus chinensis BAD83655.1
Aethina tumida XP_019881963.1
Leptinotarsa decemlineata XP_023015287.1
Diabrotica virgifera AAG23133.1
Anthonomus grandis AAN77139.1
Callosobruchus chinensis ANC73389.1
Zabrotes subfasciatus AAF73435.1
Tribolium castaneum AGW27506.1
Tribolium castaneum AGW27505.1
Tribolium castaneum XP_969234.1
Tribolium castaneum NP_001107848.1
Blaps mucronata AAO14612.1
Tribolium castaneum AAA03708.1
Tribolium castaneum AGW27504.1
Diabrotica virgifera AAF20998.1
Tribolium castaneum AAA03709.1
Aethina tumida XP_019881962.1
Tribolium castaneum XP_975485.1
Tenebrio molitor 1VIW:A|PDBID|
Tenebrio molitor 1TMQ:A|PDBID|
Phaedon cochleariae O97396.1
Agrilus planipennis XP_018335150.1
Onthophagus taurus XP_022909394.1
Nicrophorus vespilloides XP_017774160.1
Spodoptera frugiperda AAO13754.1


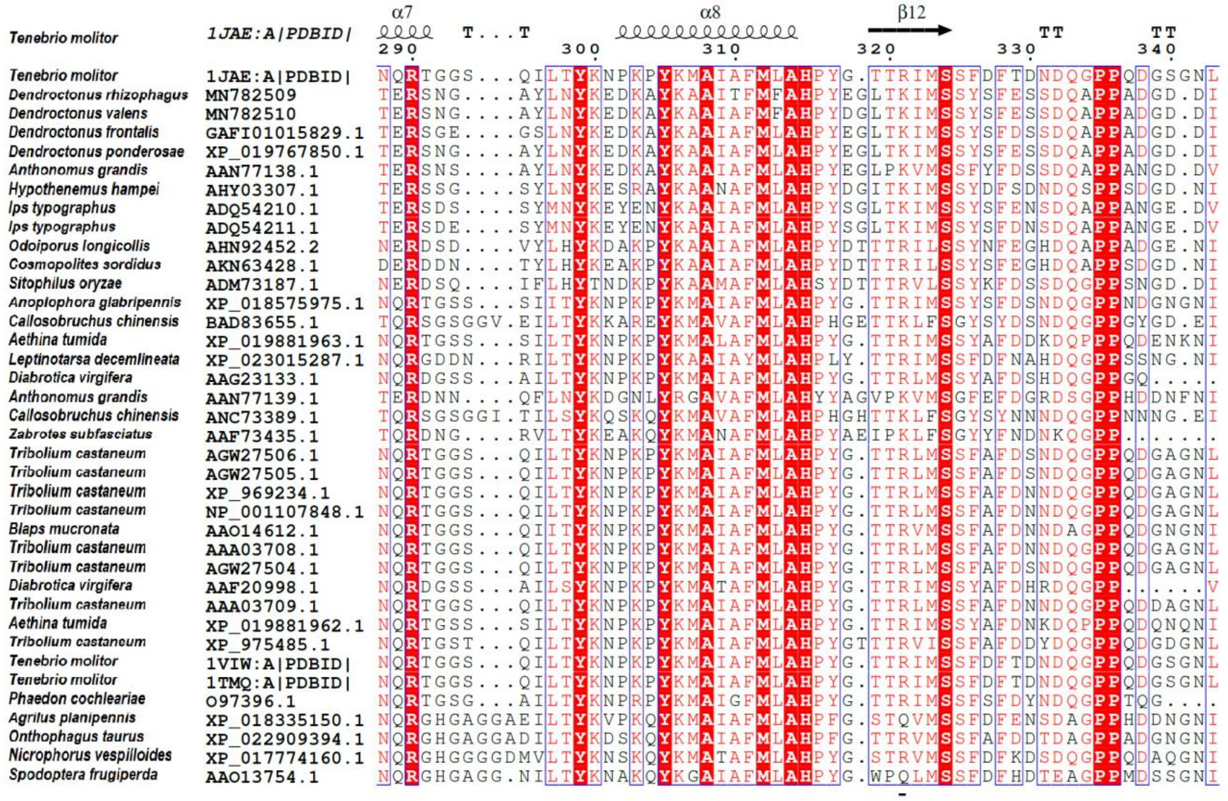

Tenebrio molitor 1JAE:A|PDBID|
α7 T...T α8 β12 TT TT
290 300 310 320 330 340
Tenebrio molitor 1JAE:A|PDBID|
Dendroctonus rhizophagus MN782509
Dendroctonus valens MN782510
Dendroctonus frontalis GAFI01015829.1
Dendroctonus ponderosae XP_019767850.1
Anthonomus grandis AAN77138.1
Hypothenemus hampei AHY03307.1
Ips typographus ADQ54210.1
Ips typographus ADQ54211.1
Odoiporus longicollis AHN92452.2
Cosmopolites sordidus AKN63428.1
Sitophilus oryzae ADM73187.1
Anoplophora glabripennis XP_018575975.1
Callosobruchus chinensis BAD83655.1
Aethina tumida XP_019881963.1
Leptinotarsa decemlineata XP_023015287.1
Diabrotica virgifera AAG23133.1
Anthonomus grandis AAN77139.1
Callosobruchus chinensis ANC73389.1
Zabrotes subfasciatus AAF73435.1
Tribolium castaneum AGW27506.1
Tribolium castaneum AGW27505.1
Tribolium castaneum XP_969234.1
Tribolium castaneum NP_001107848.1
Blaps mucronata AAO14612.1
Tribolium castaneum AAA03708.1
Tribolium castaneum AGW27504.1
Diabrotica virgifera AAF20998.1
Tribolium castaneum AAA03709.1
Aethina tumida XP_019881962.1
Tribolium castaneum XP_975485.1
Tenebrio molitor 1VIW:A|PDBID|
Tenebrio molitor 1TMQ:A|PDBID|
Phaedon cochleariae O97396.1
Agrilus planipennis XP_018335150.1
Onthophagus taurus XP_022909394.1
Nicrophorus vespilloides XP_017774160.1
Spodoptera frugiperda AAO13754.1

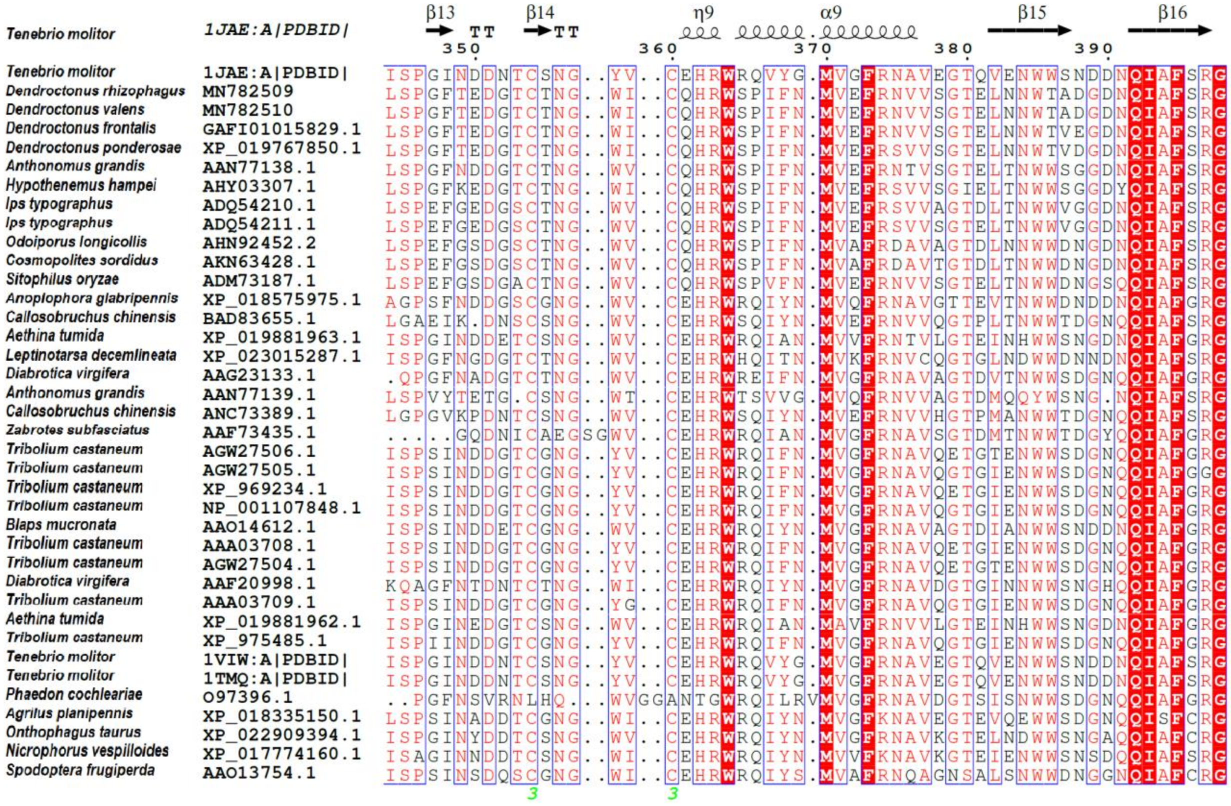
Tenebrio molitor 1JAE:A|PDBID|
β13 TT β14 TT η9 α9 β15 β16
350 360 370 380 390
Tenebrio molitor 1JAE:A|PDBID|
Dendroctonus rhizophagus MN782509
Dendroctonus valens MN782510
Dendroctonus frontalis GAFI01015829.1
Dendroctonus ponderosae XP_019767850.1
Anthonomus grandis AAN77138.1
Hypothenemus hampei AHY03307.1
Ips typographus ADQ54210.1
Ips typographus ADQ54211.1
Odoiporus longicollis AHN92452.2
Cosmopolites sordidus AKN63428.1
Sitophilus oryzae ADM73187.1
Anoplophora glabripennis XP_018575975.1
Callosobruchus chinensis BAD83655.1
Aethina tumida XP_019881963.1
Leptinotarsa decemlineata XP_023015287.1
Diabrotica virgifera AAG23133.1
Anthonomus grandis AAN77139.1
Callosobruchus chinensis ANC73389.1
Zabrotes subfasciatus AAF73435.1
Tribolium castaneum AGW27506.1
Tribolium castaneum AGW27505.1
Tribolium castaneum XP_969234.1
Tribolium castaneum NP_001107848.1
Blaps mucronata AAO14612.1
Tribolium castaneum AAA03708.1
Tribolium castaneum AGW27504.1
Diabrotica virgifera AAF20998.1
Tribolium castaneum AAA03709.1
Aethina tumida XP_019881962.1
Tribolium castaneum XP_975485.1
Tenebrio molitor 1VIW:A|PDBID|
Tenebrio molitor 1TMQ:A|PDBID|
Phaedon cochleariae O97396.1
Agrilus planipennis XP_018335150.1
Onthophagus taurus XP_022909394.1
Nicrophorus vespilloides XP_017774160.1
Spodoptera frugiperda AAO13754.1
3 3

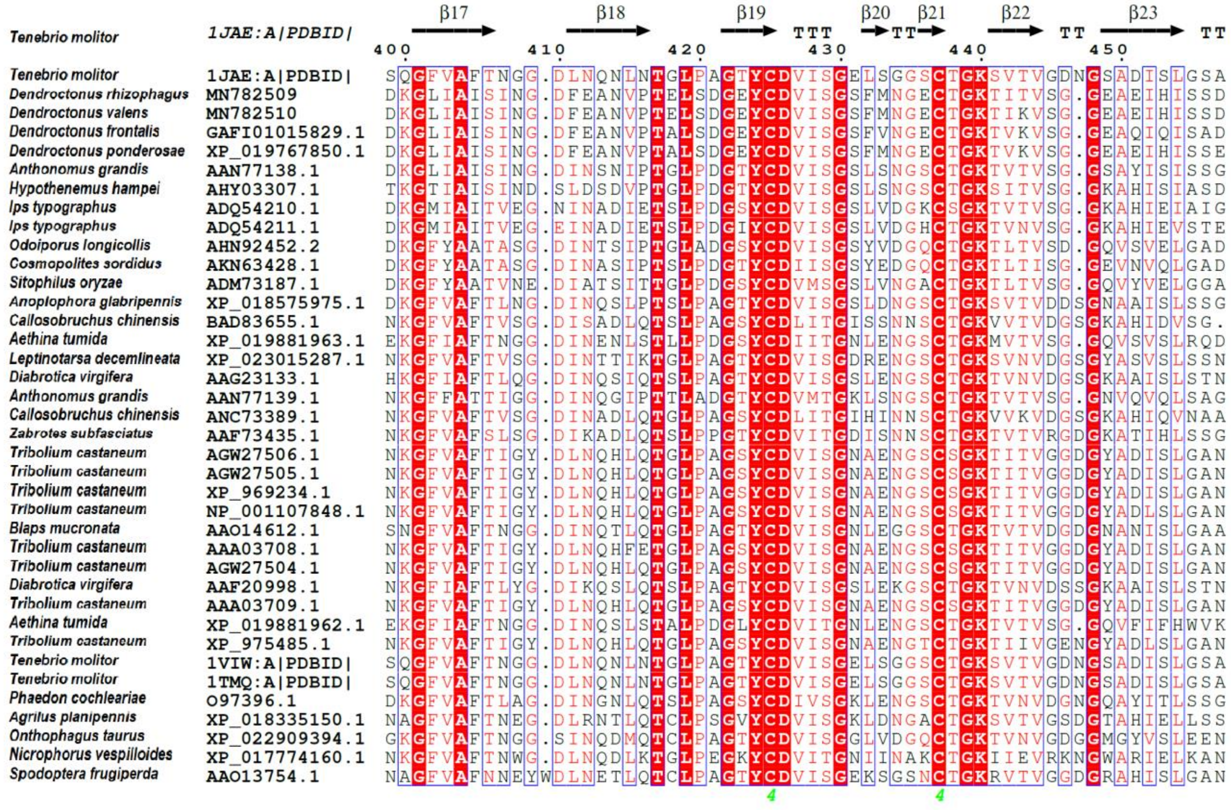
Tenebrio molitor 1JAE:A|PDBID|
β17 β18 β19 TTT β20 TT β21 β22 TT β23 TT
400 410 420 430 440 450
Tenebrio molitor 1JAE:A|PDBID|
Dendroctonus rhizophagus MN782509
Dendroctonus valens MN782510
Dendroctonus frontalis GAFI01015829.1
Dendroctonus ponderosae XP_019767850.1
Anthonomus grandis AAN77138.1
Hypothenemus hampei AHY03307.1
Ips typographus ADQ54210.1
Ips typographus ADQ54211.1
Odoiporus longicollis AHN92452.2
Cosmopolites sordidus AKN63428.1
Sitophilus oryzae ADM73187.1
Anoplophora glabripennis XP_018575975.1
Callosobruchus chinensis BAD83655.1
Aethina tumida XP_019881963.1
Leptinotarsa decemlineata XP_023015287.1
Diabrotica virgifera AAG23133.1
Anthonomus grandis AAN77139.1
Callosobruchus chinensis ANC73389.1
Zabrotes subfasciatus AAF73435.1
Tribolium castaneum AGW27506.1
Tribolium castaneum AGW27505.1
Tribolium castaneum XP_969234.1
Tribolium castaneum NP_001107848.1
Blaps mucronata AAO14612.1
Tribolium castaneum AAA03708.1
Tribolium castaneum AGW27504.1
Diabrotica virgifera AAF20998.1
Tribolium castaneum AAA03709.1
Aethina tumida XP_019881962.1
Tribolium castaneum XP_975485.1
Tenebrio molitor 1VIW:A|PDBID|
Tenebrio molitor 1TMQ:A|PDBID|
Phaedon cochleariae O97396.1
Agrilus planipennis XP_018335150.1
Onthophagus taurus XP_022909394.1
Nicrophorus vespilloides XP_017774160.1
Spodoptera frugiperda AAO13754.1
4 4

| Species | Accession | Sequence |
|---|---|---|
| *Tenebrio molitor* | *1JAE:A\|PDBID\|* | β24 TT |
| | | 460 470 |
| *Tenebrio molitor* | 1JAE:A\|PDBID\| | EDDGVLAIHVNAKL....... |
| *Dendroctonus rhizophagus* | MN782509 | DTEAAIAIHVNAKL....... |
| *Dendroctonus valens* | MN782510 | DTEAAIAIHVNAKL....... |
| *Dendroctonus frontalis* | GAFI01015829.1 | DSEAAVAIHVNAKL....... |
| *Dendroctonus ponderosae* | XP_019767850.1 | DTEAAIAIHANAKL....... |
| *Anthonomus grandis* | AAN77138.1 | DTNAAVAIHVNAKC....... |
| *Hypothenemus hampei* | AHY03307.1 | DREAAVAIHANAKL....... |
| *Ips typographus* | ADQ54210.1 | ESETAVAIHVNAKL....... |
| *Ips typographus* | ADQ54211.1 | ESECVVAVHVNAKL....... |
| *Odoiporus longicollis* | AHN92452.2 | ETEAAVAIHVNAKL....... |
| *Cosmopolites sordidus* | AKN63428.1 | ESEAAVAIHVNAKL....... |
| *Sitophilus oryzae* | ADM73187.1 | ELEAAVAIHVNAKL....... |
| *Anoplophora glabripennis* | XP_018575975.1 | EDDGVLAIHVNAKL....... |
| *Callosobruchus chinensis* | BAD83655.1 | .VDGVLAIHVNSRLQSKL... |
| *Aethina tumida* | XP_019881963.1 | EEDGVMAIHLGAKL....... |
| *Leptinotarsa decemlineata* | XP_023015287.1 | GDDFVLAIHEESKL....... |
| *Diabrotica virgifera* | AAG23133.1 | EDDGVVAIHVNAKL....... |
| *Anthonomus grandis* | AAN77139.1 | GDEIAMAIHVESKLS...... |
| *Callosobruchus chinensis* | ANC73389.1 | .LDGALAIHVNARVQSKL... |
| *Zabrotes subfasciatus* | AAF73435.1 | EPDGILAIHVSAKLTSKL... |
| *Tribolium castaneum* | AGW27506.1 | EDDGVIAIHVNAKL....... |
| *Tribolium castaneum* | AGW27505.1 | EDDGVIAIHVNAKL....... |
| *Tribolium castaneum* | XP_969234.1 | EDDGVIAIHVNAKL....... |
| *Tribolium castaneum* | NP_001107848.1 | EDDGVIAIHVNAKL....... |
| *Blaps mucronata* | AAO14612.1 | EDDGVLAIHINAKV....... |
| *Tribolium castaneum* | AAA03708.1 | EDDGVIAIHVNAKL....... |
| *Tribolium castaneum* | AGW27504.1 | EDDGVIAIHVNAKL....... |
| *Diabrotica virgifera* | AAF20998.1 | EEDGVVAIHVNAKL....... |
| *Tribolium castaneum* | AAA03709.1 | EDDGVIAIHVNAKL....... |
| *Aethina tumida* | XP_019881962.1 | MRRMEF............... |
| *Tribolium castaneum* | XP_975485.1 | EDDGVIAIHVNAKL....... |
| *Tenebrio molitor* | 1VIW:A\|PDBID\| | EDDGVLAIHVNAKL....... |
| *Tenebrio molitor* | 1TMQ:A\|PDBID\| | EDDGVLAIHVNAKL....... |
| *Phaedon cochleariae* | O97396.1 | EDDGFLAIHVGAKV....... |
| *Agrilus planipennis* | XP_018335150.1 | EDDGVLAIHINAKQS...... |
| *Onthophagus taurus* | XP_022909394.1 | EDDGVLAIHVNAKL....... |
| *Nicrophorus vespilloides* | XP_017774160.1 | EEDGVLAIHTNARL....... |
| *Spodoptera frugiperda* | AAO13754.1 | EFDMVLAIHTGPEVRIFVALS |
