# Supplementary material for: The Bark Beetle Dendroctonus rhizophagus (Curculionidae: Scolytinae) Has Digestive Capacity to Degrade Complex Substrates: Functional Characterization and Heterologous Expression of an α-Amylase
Source: Int J Mol Sci. 2020 Dec 22;22(1):36. doi: 10.3390/ijms22010036 (PMC7792934; doi:10.3390/ijms22010036)
Supplement: Supplementary file 1 [file ijms-22-00036-s001.zip › Table S1.docx]

**Table S1.** TaqMan probes

| **Assay ID** | **Assay Name** | **Reporter 1 Dye** | **Reporter 1 Quencher** | **Forward Primer Sequence** | **Reverse Primer Sequence** | **Reporter 1 Sequence** |
| --- | --- | --- | --- | --- | --- | --- |
| APAACGW | GLY3_DR | FAM | NFQ | TCACCACCATTGAAAAGGCTTCT | AGCAGATGGTGCTGAGATAATAACTTT | CCACCCTCCAAATGAG |
| APDJYMR | AMY_DR | FAM | NFQ | TCGATGCCGCAAAACATATGTG | CTTCGGAGAAAAACTCGGTGTTTAAAT | CCCGCCGATTTATCCG |
