# Supplementary material for: The Bark Beetle Dendroctonus rhizophagus (Curculionidae: Scolytinae) Has Digestive Capacity to Degrade Complex Substrates: Functional Characterization and Heterologous Expression of an α-Amylase
Source: Int J Mol Sci. 2020 Dec 22;22(1):36. doi: 10.3390/ijms22010036 (PMC7792934; doi:10.3390/ijms22010036)
Supplement: Supplementary file 1 [file ijms-22-00036-s001.zip › Table S2.docx]

**Table S2**. Minimum Information for Publication of Quantitative Real-Time PCR Experiments (MIQE) checklist.

| **Item to check** | **Details** |
| --- | --- |
| Experimental design |  |
| Definition of experimental and control groups | yes, included in the manuscript |
| Number within each group | yes, included in the manuscript |
| Assay carried out by the core or investigator’s laboratory? | yes, all experiments were performed in our laboratory |
| Acknowledgment of authors’ contributions | does not apply |
| Sample |  |
| Description | yes, included in the manuscript |
| Volume/mass of sample processed | yes, included in the manuscript |
| Microdissection or macrodissection | yes, included in the manuscript |
| Processing procedure | yes, included in the manuscript |
| If frozen, how and how quickly? | this does not apply |
| If fixed, with what and how quickly? | this does not apply |
| Sample storage conditions and duration (especially for FFPEb samples) | this does not apply |
| Nucleic acid extraction |  |
| Procedure and/or instrumentation | yes, we followed the manufacturer’s protocol without modification |
| Name of kit and details of any modifications | yes, included in the manuscript. We used a commercial RiboPure^TM^ kit (Ambion by Life Technologies, Cat. No. AM1924) |
| Source of additional reagents used | this does not apply, as we did not used any other additional reagent |
| Details of DNase or RNase treatment | we did not perform DNase or RNase treatment |
| Contamination assessment (DNA or RNA) | this was evaluated by means of A_260_/_A280_ absorbance |
| Nucleic acid quantification |  |
| Instrument and method | yes, this is included in the manuscript, and was done by means of spectrophotometry |
| Purity (A260/A280) | yes, this was evaluated |
| Yield | yes, we described the yield estimation in the manuscript |
| RNA integrity: method/instrument | yes, we described the yield estimation in the manuscript |
| RIN/RQI or Cq of 3´ and 5´ transcripts | yes, included in the manuscript |
| Electrophoresis traces | yes, included in the manuscript |
| Inhibition testing (Cq dilutions, spike, or other) | this was not evaluated |
| Reverse transcription |  |
| Complete reaction conditions | yes, we followed all manufacturer’s protocols with no single modification |
| Amount of RNA and reaction volume | yes, included in the manuscript |
| Priming oligonucleotide (if using GSP) and concentration | this does not apply |
| Reverse transcriptase and concentration | the information is not provided by the manufacturer |
| Temperature and time | yes, included in the manuscript |
| Manufacturer of reagents and catalogue numbers | yes, included in the manuscript. We used a commercial High Capacity RNA to cDNA kit (Applied Biosystems, USA; Cat. No. 4387406) |
| C_q_s with and without reverse transcription | yes, included in the manuscript |
| Storage conditions of cDNA | yes, included in the manuscript |
| qPCR target information |  |
| Gene symbol | yes, included in the manuscript |
| Sequence accession number | yes, included in the supplementary data |
| Location of amplicon | yes, included in the supplementary data |
| Amplicon length | yes, included in the supplementary data |
| In silico specificity screen (BLAST, and so on) | yes, this was done, and this information is available upon request |
| Pseudogenes, retropseudogenes, or other homologs? | does not apply |
| Sequence alignment | yes, this was done, and this information is available upon request |
| Secondary structure analysis of amplicon | yes, this was done, and this information is available upon request |
| Location of each primer by exon or intron (if applicable) | does not apply |
| What splice variants are targeted? | does not apply |
| qPCR oligonucleotides |  |
| Primer sequences | yes, included in the manuscript |
| RTPrimerDB identification number | yes, included in the supplementary data |
| Probe sequences | yes, included in the supplementary data |
| Location and identity of any modifications | does not apply |
| Manufacturer of oligonucleotides | yes, included in the manuscript |
| Purification method | the information provided by the manufacturer indicates that oligonucleotides are purified via Transgenomic Wave HPLC. The method is proprietary |
| qPCR protocol |  |
| Complete reaction conditions | yes, included in the manuscript |
| Reaction volume and amount of cDNA/DNA | yes, included in the manuscript |
| Primer, (probe), Mg^2+^, and dNTP concentrations | yes, concentration of the primers and probe are in the manuscript; however, the concentration of the master mix is not provided by the manufacturer |
| Polymerase identity and concentration | AmpliTaq Gold® DNA polymerase, UP; dNTPs with dUTP; ROX^TM^ passive reference; optimized buffer components. |
| Buffer/kit identity and manufacturer | this is included along with the PCR master mix II (Cat No. 4427788), but the information is not provided by the manufacturer |
| Exact chemical composition of the buffer | this is included along with the PCR master mix II (Cat No. 4427788), but the information is not provided by the manufacturer |
| Additives (SYBR Green I, DMSO, and so.forth) | does not apply |
| Manufacturer of plates/tubes and catalog number | only 48-well plates were used (MicroAmp^TM^, Cat. No.4375816) and optical adhesive film (MicroAmp^TM^, Cat. No. 4375928) (Applied Biosystems, USA) |
| Complete thermocycling parameters | yes, included in the manuscript |
| Reaction setup (manual/robotic) | yes, included in the manuscript |
| Manufacturer of qPCR instrument | yes, included in the manuscript |
| qPCR validation |  |
| Evidence of optimization (from gradients) | yes, included in the manuscript |
| Specificity (gel, sequence, melt, or digest) | yes, this is described in the manuscript |
| For SYBR Green I, Cq of the NTC | does not apply |
| Calibration curves with slope and *y* intercept | yes, available upon request |
| PCR efficiency calculated from slope | yes, available upon request |
| CIs for PCR efficiency or SE | yes, available upon request |
| r^2^ of calibration curve | yes, available upon request. R^2^ value was 0.99 |
| Linear dynamic range | yes, they are available upon request |
| C_q_ variation at LOD | The qPCR performed in this study did not employ a standard gene. However, we may provide our efficiency curve and dynamic range of the assay (therefore, the LOD point and SD of C_t_ at LOD) upon request |
| CIs throughout range | yes, available upon request |
| Evidence for LOD | yes, validation plots are available upon request |
| If multiplex, efficiency and LOD of each assay | does not apply |
| Data analysis |  |
| qPCR analysis program (source, version) | yes, included in the manuscript. The program used for the analysis was Microsoft Excel 2010 ver. 2011. |
| Method of Cq determination | yes, included in the manuscript |
| Outlier identification and disposition | Outliers were automatically identified by the qPCR instrument software. Once this, they were eliminated from the analysis. However, when only one out of three group replicates were eliminated, the remaining two were subsequently used in the analysis |
| Results for NTCs | yes, this is described in the manuscript |
| Justification of number and choice of.reference genes | does not apply |
| Description of normalization method | yes, included in the manuscript |
| Number and concordance of biological replicates | yes, included in the manuscript |
| Number and stage (reverse transcription or qPCR) of technical replicates | yes, included in the manuscript |
| Repeatability (intraassay variation) | yes, we estimated this as SD for the C_q_ variance and are available upon request |
| Reproducibility (interassay variation, CV) | this was not estimated |
| Power analysis | yes, included in the manuscript and the supplementary material |
| Statistical methods for results significance | yes, included in the manuscript |
| Software (source, version) | yes, included in the manuscript. The program used for the analysis was Past v. 3.26. |
| C_q_ or raw data submission with RDML | this was not reported |
